# Calcium, cobalt, strontium and cerium-based binary silicate bioactive nanoglasses: rational comparison in bioactive properties for optimizing tissue repair applications

**DOI:** 10.1093/rb/rbag004

**Published:** 2026-03-01

**Authors:** Yanzi Zhao, Jing Tian, Liuyang Zhang, Long Zhang, Qian Huang, Xiaoyan Qu, Bo Lei

**Affiliations:** Key Laboratory of Shaanxi Province for Craniofacial Precision Medicine Research, College of Stomatology, Xi’an Jiaotong University, Xi’an 710054, China; Frontier Institute of Science and Technology, Xi’an Jiaotong University, Xi’an 710054, China; Frontier Institute of Science and Technology, Xi’an Jiaotong University, Xi’an 710054, China; Frontier Institute of Science and Technology, Xi’an Jiaotong University, Xi’an 710054, China; Department of Respiratory and Critical Care Medicine, The Second Affiliated Hospital of Xi’an Jiaotong University, Xi’an 710004, China; Key Laboratory of Shaanxi Province for Craniofacial Precision Medicine Research, College of Stomatology, Xi’an Jiaotong University, Xi’an 710054, China; Frontier Institute of Science and Technology, Xi’an Jiaotong University, Xi’an 710054, China; Key Laboratory of Shaanxi Province for Craniofacial Precision Medicine Research, College of Stomatology, Xi’an Jiaotong University, Xi’an 710054, China; Frontier Institute of Science and Technology, Xi’an Jiaotong University, Xi’an 710054, China; Department of Respiratory and Critical Care Medicine, The Second Affiliated Hospital of Xi’an Jiaotong University, Xi’an 710004, China; Department of Orthopedics, The First Affiliated Hospital of Xi’an Jiaotong University, Xi’an 710061, China

**Keywords:** bioactive materials, bioactive glass nanoparticles, ion doping, wound repair

## Abstract

Silicate bioactive glass nanoparticles (SBGNs) are promising for tissue repair, but multi-metals doped systems often suffer from compositional complexity, undefined ion-function relationships, and insufficient mechanistic validation. Herein, we established a compositionally simplified binary platform and synthesized four single-ion doped SBGNs (MBGNs: BCa, BCo, BSr, BCe) incorporating representative alkaline-earth, transition, and rare-earth elements. Their tissue repair-relevant bioactivities were systematically compared under unified synthesis and evaluation conditions. All MBGNs exhibited good cytocompatibility but displayed distinct, element-dependent bioactivity profiles. BCa enhanced baseline bioactivity by promoting apatite nucleation in simulated body fluid. BSr showed the strongest anti-inflammatory activity, reducing *Il6* and *Il1β* expression by over 50% and facilitating M2-like macrophage polarization. BCe showed pronounced antioxidant activity, degrading 81.6% of H_2_O_2_ and eliminating 90% of intracellular reactive oxygen species (ROS) via CeO_2_-mediated redox cycling. BCo potently promoted angiogenesis, upregulating *VEGF* and *CD31* expression by up to 2.3- and 1.8-fold, and enhancing cell migration and tubular formation. In acute and bacterial infected full-thickness wound models, BSr and BCe accelerated wound closure by 20–40% versus undoped BSi and 3M dressings and more effectively restored vasculature and hair follicles, with BSr exhibiting the greatest healing efficacy. Dose-optimized BCo enhanced hair-follicle regeneration by 123% but was limited by a narrow biosafety window, whereas BCa reduced epidermal thickness by 38% while otherwise resembling BSi. Overall, these data delineate a clear hierarchy of tissue-repair efficacy (BSr > BCe > BCo > BCa ≈ BSi) and show that compositional simplification enables ion-guided design and functional tuning of SBGNs, providing a concise framework for developing simpler yet more potent biomaterials for tissue repair.

## Graphical abstract

**Figure:**
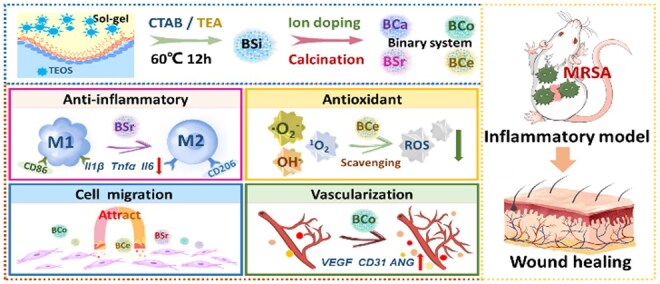

## Introduction

Bioactive glass (BG), an inorganic amorphous biomaterial extensively studied for its capacity to form stable interfaces with both soft and hard tissues, has been widely used in bone, dental and skin repair [1]. Compared with conventional BGs, nanosized BGs have become robust model systems for dissecting composition-structure-function relationships, owing to their tailorable chemistry and highly reactive interfaces [2]. Among these, silicate bioactive glass nanoparticles (SBGNs) integrate structural stability with controlled degradation, enabling the sustained release of bioactive ions (Si/Ca/P). These ions can activate key signaling pathways, including hypoxia-inducible factor-1α (HIF-1α), promote angiogenesis, and modulate inflammatory mediators such as tumor necrosis factor-α (*Tnfα*) and interleukin-6 (*Il6*), thereby establishing a programmable ionic microenvironment that supports tissue regeneration [3–5]. In contrast, faster-degrading borate- and borosilicate-based BGNs, which possess loosely crosslinked BO_3_/BO_4_^-^rich networks, undergo rapid dissolution and burst-like ion release (e.g. B^3+^, Ca^2+^). This accelerated release can promote early hemostasis, antibacterial activity, angiogenesis, and granulation tissue formation. However, their rapid disintegration is often accompanied by pronounced fluctuations in local ion concentrations and pH, thereby disrupting the continuity of biological functions and tissue remodeling [6–8]. Consequently, SBGNs, with a more controlled ion-release profile that better preserves microenvironmental homeostasis, provide a more suitable platform for dissecting quantifiable biological effects and mechanisms in tissue repair.

Building on the stable ionic background provided by SBGNs, rational selection and controlled doping of metal ions is regarded as an effective strategy to enhance their multifunctionality and overall biological performance. Previous studies have demonstrated that strontium and zinc-doped SBGNs possess pronounced anti-inflammatory activity and can accelerate wound closure [9, 10]. SBGNs modified with manganese and cerium exhibit notable antioxidant effects, reducing cellular oxidative stress levels [11, 12]. Copper-containing SBGNs have been shown to stimulate cytokine secretion from human umbilical vein endothelial cells, consequently promoting vascularization and deposition of extracellular matrix proteins [13]. In recent years, researchers have primarily focused on ternary SBGNs (SiO_2_-CaO-P_2_O_5_, SiO_2_-CaO-MgO), quaternary SBGNs (SiO_2_-CaO-P_2_O_5_-MeO), and even quintuple bioactive glasses for various applications. For instance, the ternary mesoporous SBGNs was used for surgical hemostasis [14], while the quaternary SBGNs doped with europium, strontium, or zinc for chemotherapy and postoperative oncological wound repair [6, 15]. The quintuple SBGNs co-doped with cerium and gadolinium were employed for visual wound repair [16]. These nanoparticles (NPs) have exhibited favorable hemostatic, anti-inflammatory, antioxidant, and pro-angiogenic effects. However, such high-dimensional compositions hinder mechanistic analysis: the contributions of hydrolysis-derived silicate species and multiple metal ions are difficult to distinguish, and their possible synergistic or antagonistic effects are rarely quantified under unified criteria, reducing the reproducibility and interpretability of reported findings. Therefore, we designed compositionally simplified binary systems with single metal-ion doping and evaluated them under standardized synthesis and testing conditions to clarify their biological activities and underlying mechanisms.

Currently, there are a limited number of studies on binary SBGNs, with a primary focus on tissue repair and bone tissue engineering. Notably, Wang *et al*. [17] reported SiO_2_-CaO NPs, which effectively promote wound coagulation. Compared with multicomponent systems, binary formulations have a simpler composition, making it easier to focus on evaluating the biological effects of a single therapeutic ion under comparable conditions. The four representative elements—calcium (Ca), strontium (Sr), cobalt (Co), and cerium (Ce) that are highly relevant to the tissue repair process were selected for dope silica. Calcium ions (Ca^2+^) play a crucial role in cellular signal transduction by activating pathways such as NF-κB and MAPK, which are essential for inflammation regulation and cell proliferation [18]. Strontium ions (Sr^2+^) modulate macrophage polarization, shifting the inflammatory response toward an anti-inflammatory phenotype by suppressing the expression of *Tnfα* and *Il6* [10, 19]. Cobalt ions (Co^2+^) stabilize hypoxia-inducible factor-1α (HIF-1α), thereby promoting endothelial cell migration, proliferation, and angiogenesis [20]. Cerium ions (Ce^3+^/Ce^4+^) mitigate oxidative stress while enhancing vascularization and accelerating tissue repair [21]. The four elements mentioned above represent different categories of bioactive metals: alkaline earth metals (Ca, Sr), transition metals (Co) and rare earth elements (Ce), facilitating the establishment of distinguishable ‘ion-function’ relationships.

In this study, we developed the binary bioactive glass nanoparticles (MBGNs) by incorporating four metal elements: calcium, strontium, cobalt and cerium, and the detailed bioactive properties were systematically compared. Initially, the mesoporous silica nanoparticles (BSi) were prepared via a sol-gel-template method. Subsequently, a solid-state reaction was employed to incorporate Ca, Co, Sr and Ce into the silica network, resulting in the formation of four BGN systems: BCa (70 mol% SiO_2_-30 mol% CaO), BCo (70 mol% SiO_2_-30 mol% CoO), BSr (70 mol% SiO_2_-30 mol% SrO) and BCe (70 mol% SiO_2_-30 mol% Ce_2_O_3_/CeO_2_). This study primarily investigates the biological properties of these materials relevant to cutaneous wound healing, including biocompatibility, anti-inflammatory and antioxidant activities, as well as pro-angiogenic capacity. Furthermore, it systematically analyzed the relationship between their physicochemical structures and biological functions, and further evaluated their therapeutic performance in both acute inflammatory wounds and methicillin-resistant *Staphylococcus aureus* (MRSA)-infected inflammatory wound models.

## Materials and methods

### Preparation and characterizations of BSi and MBGNs

MBGNs were prepared by a combination of sol-gel and solid-state reaction methods. First, BSi was synthesized as the silicon-based precursor. The specific steps are as follows: 1.2 g of cetyltrimethylammonium bromide (CTAB) and 36 μL of triethanolamine (TEA) were dissolved in 10.8 mL of deionized water and stirred at 60 °C at 300  rpm for 1 h. Subsequently, 1.2 mL of tetraethyl orthosilicate (TEOS) mixed with 4.8 mL of cyclohexane was slowly added dropwise into the above solution in three portions at 15-min intervals. The mixture was further stirred at 60 °C for 12 h to complete the hydrolysis reaction. After reaction, the white precipitate was collected by centrifugation, washed with ethanol and deionized water, and dried to obtain the silica (BSi).

Subsequently, metal ion incorporation was achieved *via* a solid-state reaction, using the cobalt-doped sample (BCo, 70 mol% SiO_2_-30 mol% CoO) as an example: 59.87 mg of Co(NO_3_)_2_ was dissolved in 10 mL anhydrous ethanol, followed by the addition of 0.1 g of uncalcined BSi. The mixture was stirred at room temperature for 6 h until complete evaporation of ethanol, allowing Co^2+^ to uniformly incorporate into the glass network. The resulting solid was then calcined at 600 °C for 5 h to obtain the BCo. Using the same ion-doping strategy, a series of other binary MBGNs were further synthesized, including 70 mol% SiO_2_-30 mol% CaO (BCa), 70 mol% SiO_2_-30 mol% SrO (BSr) and 70 mol% SiO_2_–30 mol% Ce_2_O_3_/CeO_2_ (BCe). The morphology, mesoporous characteristics, elemental distribution, crystal structure, and zeta potential of the as-prepared samples were systematically characterized using various analytical techniques. Detailed information is provided in the supplementary information (SI). In addition, their mineralization activity was evaluated, and the corresponding methods are also described in the SI. To provide an intuitive overview of the compositions and fabrication procedures of the different glass systems, the chemical compositions of each sample are summarized in Supplementary Table S1.

### Cytocompatibility and anti-inflammatory properties evaluations

Macrophages (RAW264.7), human umbilical vein endothelial cells (HUVECs) and fibroblasts (L929) were used as model cells, and cell compatibility was evaluated using an AlamarBlue assay kit. Detailed experimental procedures are provided in the SI. Subsequently, to evaluate the anti-inflammatory effects of MBGNs, RAW264.7 cells were co-cultured with 100 ng/mL lipopolysaccharide (LPS, Beyotime) at 37 °C in a 5% CO_2_ incubator for 24 h, followed by treatment with MBGNs at concentrations of 12.5 μg/mL, 25 μg/mL and 50 μg/mL, respectively. After a total incubation period of 24 h, total cellular RNA was extracted, and RT-qPCR was performed to detect gene expression levels. Specifically, the expression levels of *Tnfα*, *Il6*, and interleukin-1β (*Il1β)* were analyzed. Detailed primer information can be found in Supplementary Table S2. Finally, flow cytometry was used to evaluate macrophage phenotypic switching. RAW264.7 cells were co-cultured with 200 ng/mL of LPS for 24 h (37 °C, 5% CO_2_), followed by adding cell culture medium containing 50 μg/mL BSi, BCa, BCo, BSr and BCe NPs. Flow cytometry (CytoFLEX, Beckman Coulter) was employed to assess the expression of the M1 surface marker CD86 on RAW264.7 cells.

### Antioxidant and angiogenic properties assessments

LPS-stimulated RAW264.7 cells were used as a model, and intracellular reactive oxygen species (ROS) were visualized by fluorescence staining with a ROS detection kit (S0033S, Beyotime) and observed under a confocal laser scanning microscope (FV1200, Olympus). Detailed experimental procedures are provided in the SI. On this basis, the catalase-like (CAT-like) activity of BSi and MBGNs was evaluated using a catalase activity assay kit (S0051, Beyotime). Specifically, 2 mg of MBGNs from each group were incubated with an H_2_O_2_ solution at 37°C in the dark for 30 min. After adding the stop solution, the residual H_2_O_2_ reacted with the chromogenic substrate to generate a red product, and the absorbance was measured at 240 nm to calculate the H_2_O_2_ scavenging efficiency.

Angiogenesis-related experiments were performed using RT-qPCR and immunohistochemistry (IHC). HUVECs were incubated with the MBGNs materials for a duration of 24 h. Following the incubation, RNA was extracted from the cells and subjected to reverse transcription to generate complementary DNA (cDNA). RT-qPCR was then performed to evaluate the expression levels of angiogenesis-related genes, including vascular endothelial growth factor (*VEGF*), angiopoietin (*ANG*) and platelet endothelial cell adhesion molecule (*CD31*). Detailed primer information can be found in Supplementary Table S2. In parallel, IHC staining for VEGF protein was carried out. Hematoxylin staining was performed to visualize the nuclei. The expression of VEGF was observed as a brown signal using an orthogonal microscope (BX53, Olympus). The quantification of VEGF expression was performed using Image J software.

### Cell migration and tubular formation capacity evaluations

HUVECs were selected as the model cells for cell migration and tube formation assays. In the scratch assay, HUVECs were seeded in six-well plates and allowed to grow until reaching confluence. Subsequently, a single cell-free gap was created by carefully scratching across the well using a 10 μL pipette tip. The experimental group was then treated with DMEM supplemented with 2% fetal bovine serum (FBS) and 25 μg/mL of BSi and MBGNs, while the control group received DMEM supplemented with 2% FBS only. Cell migration in the scratched area was recorded at 0, 24 and 48 h under an inverted microscope (1X523, Olympus).

In the tubular formation assay, HUVECs were seeded in a 96-well plate pre-coated with Matrigel, and DMEM (control) or DMEM containing 25 μg/mL BSi/MBGNs (experimental) was added. After 6 h of incubation, cells were stained with calcein-AM, and tubular formation was observed and recorded under an inverted fluorescence microscope.

### *In vivo* wound healing effect assessment

The *in vivo* experiments were conducted according to the Animal Care and Use Committee (IACUC) of Xi’an Jiaotong University (No. XJTUAE2024-1199). A total of 108 female Kunming mice (8 weeks old, 30–35 g) were used to establish both acute full-thickness skin wound models and MRSA-infected wound models. The animals were randomly divided into six treatment groups (3M film, BSi, BCa, BCo, BSr, and BCe; *n* = 9).

In the acute inflammatory wound model, two symmetrical full-thickness excisional wounds with a diameter of 7 mm were created on the backs of the mice. Five milligrams of powder (BSi, BCa, BCo, BSr or BCe) was evenly sprinkled onto the wound surface of the mice in the experimental groups. Tegaderm film (3M film) was then applied to cover the wound, providing protection to the wound, preventing skin atrophy, and minimizing material shedding. The control group mice were treated with 3M film alone.

In the MRSA-infected inflammatory wound model, 7 mm full-thickness wounds were similarly generated on the dorsal skin. Subsequently, 10 μL of MRSA suspension (1 × 10^7^ CFU/mL) was applied to the wound to induce inflammation. After successful establishment of infection, 2 mg of BSi or MBGNs samples were evenly applied to the wound surface and covered with a 3M film dressing. The control group was treated with the film alone.

Wound areas were photographed on day 0 (the day of surgery), 3, 7 and 14, and the wound closure rate was quantified using ImageJ software. On day 3, 7 and 14, wound tissues were harvested and fixed in 4% paraformaldehyde, followed by hematoxylin and eosin (H&E) staining for histological analysis. Sections were imaged using an upright microscope, and ImageJ was used to quantitatively assess the width of immature granulation tissue, epidermal thickness, and the number of hair follicles.

### Statistical analysis

Each experiment was performed with a minimum of three replicates, and the resulting data are presented as the mean ± standard deviation (mean ± SD). Data analysis was conducted using GraphPad Prism 8.0 software. For comparisons between two groups, an unpaired two-tailed Student’s t-test was employed. For comparisons among three or more groups, one-way analysis of variance (ANOVA) followed by Tukey’s *post hoc* test was used to determine statistical significance. A value *P *< 0.05 (*) is considered statistically significant, indicating a significant difference between the compared groups. A more pronounced level of statistical significance is denoted by *P *< 0.01 (**), indicating a more substantial difference between the groups. The highest degree of statistical significance is represented by *P *< 0.001 (***), which signifies an extremely significant difference between the groups. Conversely, the abbreviation “ns” was used to denote no significant difference when the observed *P* values exceeded 0.05.

## Results

### Characterizations of MBGNs

**Figure 1:**
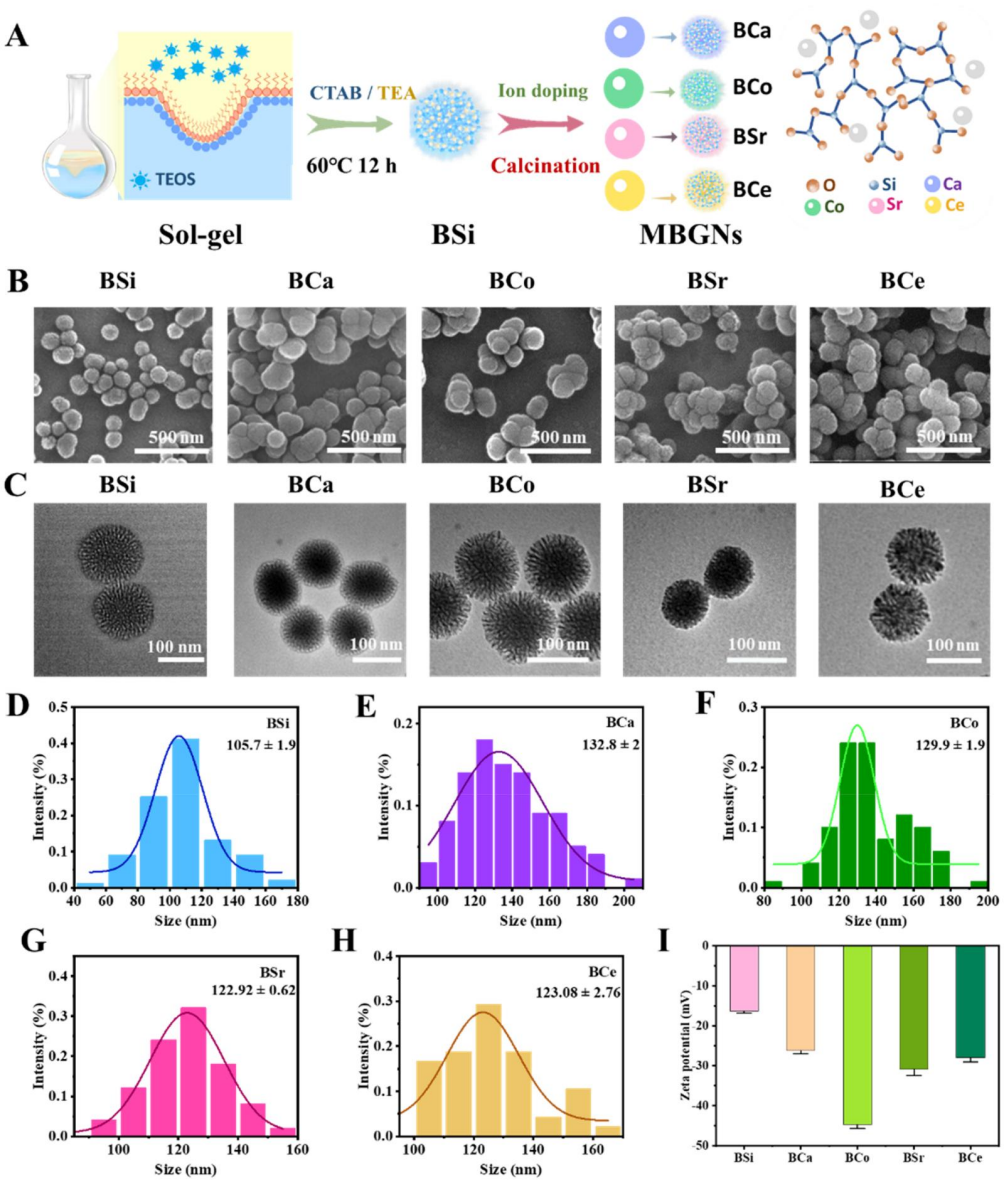
Synthesis and characterizations of BSi and MBGNs. (**A**) Schematic diagram of the formation of BSi and MBGNs (**B**) TEM image of BSi and MBGNs. (**C**) SEM image of BSi and MBGNs. Particle size distribution charts of BSi and MBGNs (**D**) BSi (**E**) BCa (**F**) BCo (**G**) BSr (**H**) BCe. (**I**) Zeta potential of BSi and MBGNs.

Figure 1 illustrates the schematic structure and morphological characterizations of samples. Based on the hydrolysis and condensation at the interface between the oil phase (TEOS/cyclohexane) and the aqueous phase (CTAB/TEA), the metal ions could be efficiently incorporated into the mesoporous silica and form binary metal-doped MBGNs after further heat treatment (BCa, BCo, BCe and BSr) (Figure 1A and Supplementary Figure S1). Scanning electron microscope (SEM) images of BSi, BCa, BCo, BSr and BCe reveal well-defined spherical NPs with relatively narrow size distributions and slightly rough surfaces (Figure 1B). Transmission electron microscope (TEM) observations further demonstrate stripe-like or worm-like mesoporous channels on the particle surfaces, confirming that all five SBGNs possess a typical mesoporous framework (Figure 1C). Based on these morphological findings, nitrogen adsorption–desorption measurements were performed to quantify the pore structure parameters (Supplementary Figure S2). All samples exhibit type IV isotherms with H3 hysteresis loops, indicative of characteristic mesoporosity. BJH analysis shows that the pore sizes of all groups fall within the 10–20 nm range, with average pore diameters of 17.02 nm, 15.46 nm, 14.90 nm and 16.43 nm for BSi, BCa, BCo and BSr, respectively, whereas BCe displays the smallest average pore size (12.08 nm). This pore shrinkage trend is consistent with the branched, dendritic surface features observed in the TEM images of BCe. Particle size statistics (Figure 1D–H) further indicate that, compared with BSi (≈105 nm, broader distribution), the MBGNs exhibit slightly increased yet more uniform average particle sizes of 132 nm, 129 nm, 122 nm and 123 nm for BCa, BCo, BSr and BCe, respectively. Zeta potential measurements further revealed their colloidal stability (Figure 1I): all samples were negatively charged in deionized water, with BSi exhibiting a zeta potential of −16.4 mV, whereas the MBGNs showed more negative values of −26.2 mV (BCa), −44.8 mV (BCo), −28.0 mV (BSr) and −30.9 mV (BCe), indicating that metal ion doping enhances the dispersion stability of the particles in aqueous media.

**Figure 2:**
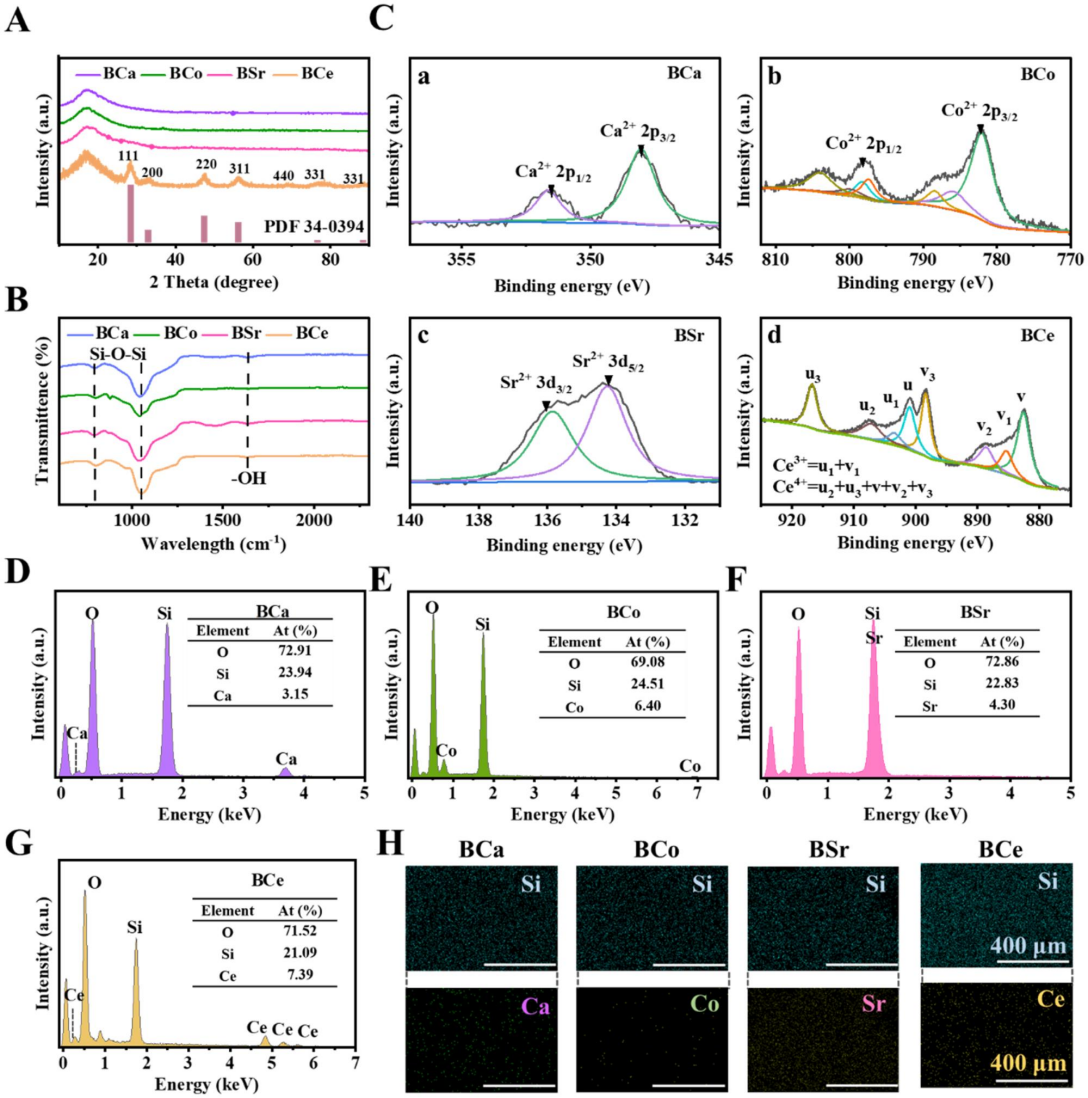
Physical and chemical structure characterization of MBGNs. (**A**) XRD pattern of MBGNs. (**B**) FTIR spectra of MBGNs. (**C**) XPS spectra of MBGNs: BCa (a), BCo (b), BSr (c), and BCe (d). (**D**) Energy spectra of BCa. (**E**) Energy spectra of BCo. (**F**) Energy spectra of BSr. (**G**) Energy spectra of BCe. (**H**) Elemental distribution image of MBGNs.

The phase structure of the MBGNs was analyzed by X-ray diffraction (XRD) (Figure 2A). The XRD patterns of BCa, BCo and BSr exhibited broad, diffuse halos without any sharp diffraction peaks, consistent with an amorphous silicate glass structure (Supplementary Figure S3A). In contrast, the XRD pattern of the Ce-doped sample displayed additional diffraction peaks at 2θ = 28.5°, 33.2°, 47.7°, 56.3°, 68.66°, 78.4° and 88.76°, corresponding to the (111), (200), (220), (311), (440), and (331) planes of crystalline CeO_2_ (PDF 34-0394), indicating that BCe possesses a composite structure with embedded CeO_2_ crystalline domains. Fourier-transform infrared spectroscopy (FTIR) was employed to further investigate the chemical structure of the MBGNs (Figure 2B). All samples exhibited characteristic absorption peaks at 800 cm^−1^ (bending vibration of Si–O–Si), 1100 cm^−1^ (symmetric stretching vibration of Si–O–Si), and 1635 cm^−1^ (stretching vibration of –OH groups from adsorbed water). After metal ion doping, the positions of these bands remained essentially unchanged, whereas the intensities of the Si-O-Si and -OH-related bands decreased (Supplementary Figure S3B), indicating that metal-ions incorporation perturbs the local silicate network without altering its overall framework. X-ray photoelectron spectroscopy (XPS) was further used to determine the oxidation states of the doped metals. The binding energies of Ca 2p_1/2_ and Ca 2p_3/2_ were measured at 351.7 eV and 348 eV, respectively, indicating that Ca was doped into the BSi glass network in the form of Ca^2+^ (Figure 2C(a)). Similarly, the binding energies of Co 2p_1/2_ and 2p_3/2_, and Sr 3d_3/2_ and 3d_5/2_ corresponded to Co^2+^ (Figure 2C(b)) and Sr^2+^ (Figure 2C(c)), respectively. The three peaks observed in BCe were assigned to the mixed valence states of Ce^3+^ and Ce^4+^ (Figure 2C(d)). Moreover, energy-dispersive X-ray spectroscopy (EDS) and elemental mapping (Figure 2D–H) reveal clear Si and O signals in all samples, together with signals corresponding to the doped metals (Ca, Co, Sr Ce). In BCa, BCo, and BSr, Ca, Co, and Sr are homogeneously distributed within the amorphous glass matrix, with no evidence of phase separation. In contrast, although partial crystallization of Ce^3+^/Ce^4+^ occurred in the BCe sample during calcination, forming CeO_2_ crystalline domains, a considerable fraction of cerium remained homogeneously dispersed within the silicate glass matrix, leading to a cooperative coexistence of crystalline and amorphous phases.

Based on the above structural and compositional analyses, the basic apatite-formed bioactivity of the five SBGNs was further evaluated (Supplementary Figure S4). After immersion in simulated body fluid (SBF) for 7 days, BSi and BCo still exhibit amorphous diffraction features, and no new crystalline phases are detected. The diffraction peaks of BCe remain identical to those at day 0, indicating that the CeO_2_ phase is structurally stable in SBF. In contrast, BCa and BSr exhibited additional reflections superimposed on the amorphous background at approximately 2θ = 25.9°, 31.7° and 46.7°, corresponding to the characteristic peaks of hydroxyapatite (HAp, PDF#09-0432), suggesting the formation of an apatite-like mineralized layer on their surfaces.

### Cytocompatibility of MBGNs *in vitro*

**Figure 3:**
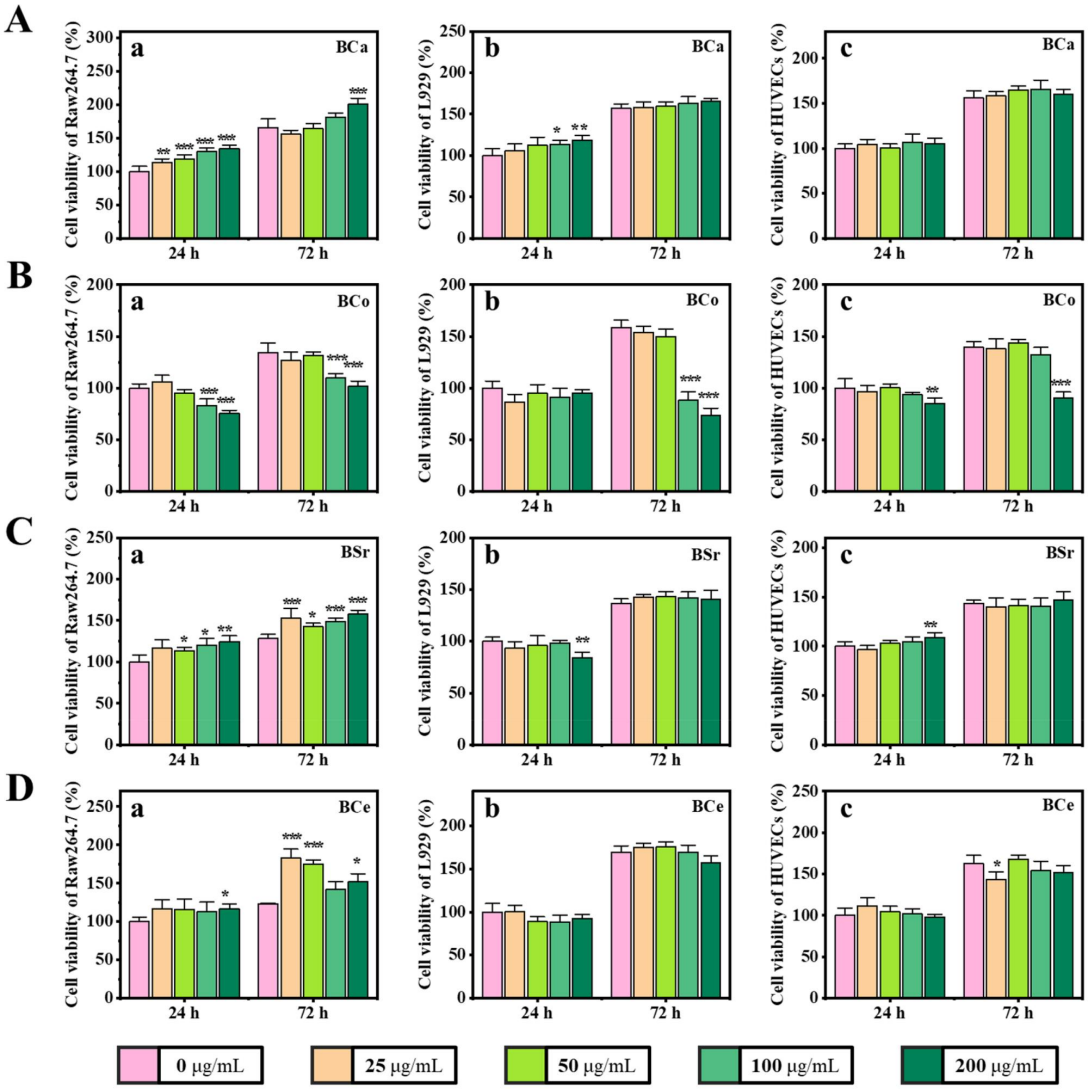
Cytocompatibility of MBGNs. (**A–D**) Cell viability of Raw264.7 (a), L929 (b), and HUVECs (c) cells after incubation with MBGNs for 24 h and 72 h: (**A**) BCa, (**B**) BCo, (**C**) BSr, and (**D**) BCe (*n* = 5, *P < 0.05, **P < 0.01, ***P < 0.001).

Cytocompatibility assays showed that BCa maintained cell viabilities above 90% for RAW264.7, L929, and HUVECs after 24 and 72 h of co-culture at 200 μg/mL (Figure 3A), and promoted RAW264.7 proliferation in a concentration-dependent manner (Figure 3A(a)). BCo did not exhibit obvious cytotoxicity in the range of 25–50 μg/mL; however, when the concentration exceeded 50 μg/mL, the viability of all three cell types decreased by approximately 25% on average, indicating dose-dependent cytotoxicity (Figure 3B). BSr showed good cytocompatibility at all tested concentrations and, at 200 μg/mL, increased the proliferation of RAW264.7 and HUVECs by about 25% and 10%, respectively (Figure 3C(a, c)). BCe induced a concentration-dependent proliferation of RAW264.7 cells, with more pronounced stimulatory effects at lower concentrations (Figure 3D(a)), whereas L929 and HUVECs showed no evident cytotoxicity or appreciable changes in proliferation (Figure 3D(b, c)). Based on these concentration-response characteristics, and to ensure comparability among materials and robustness of subsequent analyses, all subsequent *in vitro* experiments were performed at standardized concentrations of 12.5, 25 and 50 μg/mL.

Subsequently, we further evaluated the cytocompatibility of MBGN extracts (Supplementary Figure S5). Overall, the extract-based assays yielded more favorable results than the direct-contact method: BSi maintained good cytocompatibility at 2 mg/mL (Supplementary Figure S5A), and no obvious adverse effects were observed for BCa (Supplementary Figure S5B), BSr (Supplementary Figure S5D), or BCe (Supplementary Figure S5E), consistent with the trends seen in the direct-contact assays. Notably, however, the 2 mg/mL extract of BCo reduced the viability of HUVECs and RAW264.7 cells to approximately 50%, indicating that BCo also exhibits marked dose sensitivity under extract conditions (Supplementary Figure S5C).

### Anti-inflammatory properties and macrophage polarization analysis of MBGNs

**Figure 4:**
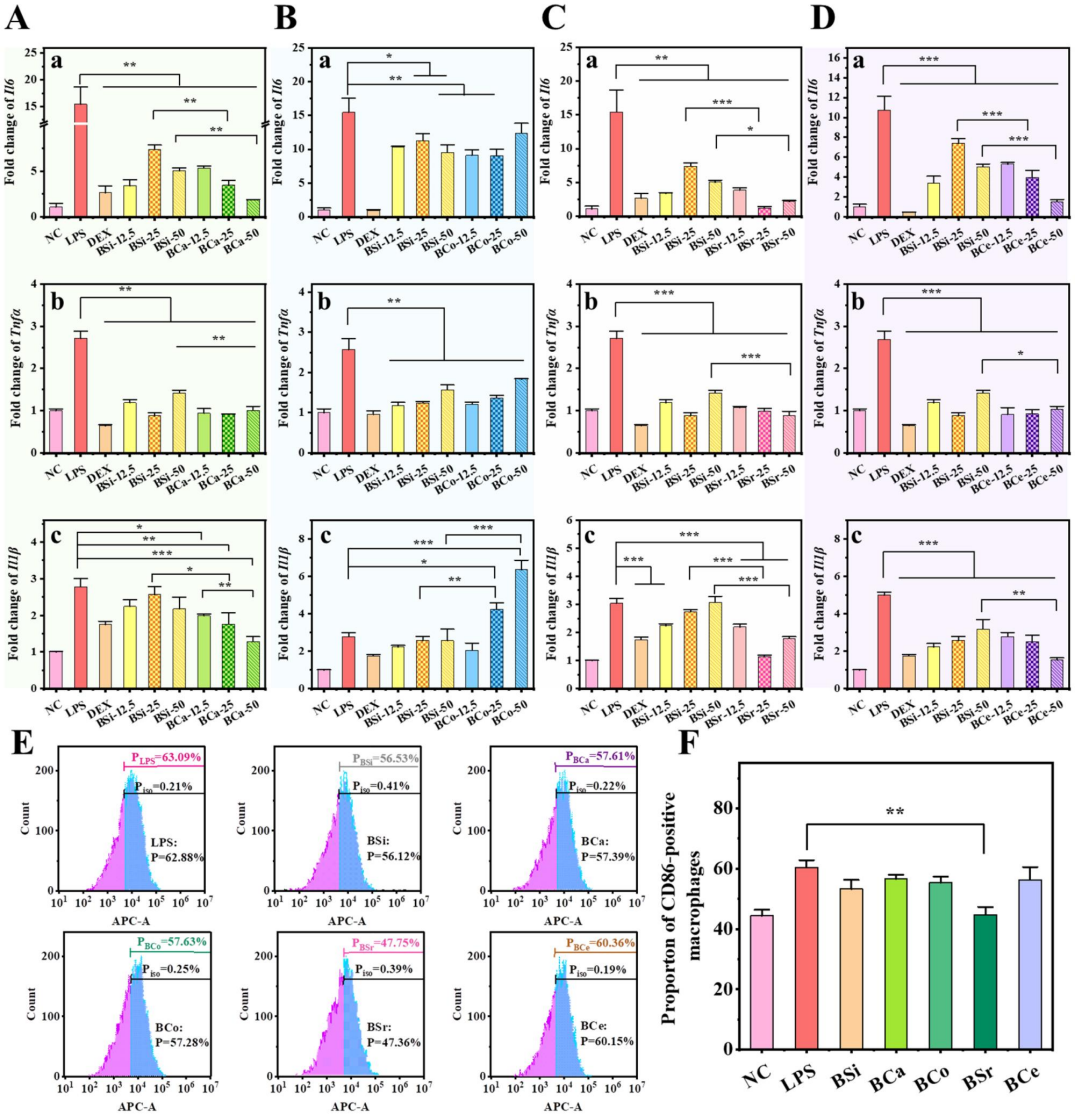
Anti-inflammatory effects and macrophage phenotype modulation capabilities of MBGNs. (**A–D**) Gene expression level of (a) *Il6*, (b) *Tnfα*, and (c) *Il1β* in RAW264.7 cells after treatment with MBGNs: (**A**) BCa, (**B**) BCo, (**C**) BSr, and (**D**) BCe (*n* = 3, *P < 0.05, **P < 0.01). (**E**) Macrophage phenotype transformation after RAW264.7 cells are treated with BSi and MBGNs. (**F**) Statistical analysis of macrophage polarization (CD86-positive proportion). (*n* = 3, **P < 0.01).

The anti-inflammatory activity of nanomaterials plays a crucial role in tissue repair by modulating cytokine expression and immune cell phenotypes to reconstruct a pro-regenerative microenvironment. As shown in Figures 4A–D, both BSi and the MBGNs downregulated the expression of inflammation-related genes to varying degrees, although the magnitude of inhibition differed among groups.

BSi exhibited a stable anti-inflammatory effect: at all tested concentrations, it reduced the expression of *Il6* and *Tnfα* (*P* < 0.01) (Figure 4A(a, b)). In particular, at 12.5 μg/mL, *Il6* and *Tnfα* levels decreased by approximately 77.9% and 56.2%, respectively, whereas *Il1β* expression was not markedly affected (Figure 4A(c)). On this basis, Ca incorporation further strengthened the anti-inflammatory response (Figure 4A). Compared with BSi at the same concentrations, BCa increased the suppression of *Il6* by 53% and 62% at 25 and 50 μg/mL (Figure 4A(a)), respectively, and additionally decreased *Il1β* expression by 31.6% and 39.4%. At 50 μg/mL (Figure 4A(c)), *Tnfα* expression in the BCa group was also reduced by 28.7% relative to BSi (Figure 4A(b)), indicating an overall dose-dependent enhancement of anti-inflammatory activity. In contrast to this trend, BCo at a low concentration (12.5 μg/mL) exerted an anti-inflammatory effect comparable to that of BSi, with no differences in the expression of inflammatory cytokines (Figure 4B). However, upon increasing the concentration, its regulatory profile changed markedly: at 25 and 50 μg/mL, *Il1β* expression in the BCo group increased by approximately 64.6% and 147%, respectively, relative to BSi at the corresponding concentrations, suggesting that Co incorporation may induce or potentiate inflammatory responses at higher doses (Figure 4B(c)). By comparison, BSr exhibited consistently stronger anti-inflammatory effects than BSi across all tested concentrations (Figure 4C). At 25 μg/mL, the expression of *Il6* and *Il1β* was reduced by 83.3% and 58.1%, respectively, relative to BSi, whereas at 50 μg/mL the corresponding reductions were 43.6% and 18.6% (Figure 4C(a, c)). In addition, *Tnfα* expression at 50 μg/mL decreased by 37.2% compared with BSi (Figure 4C(b)). Taken together, these data indicate that 25 μg/mL represents an optimal anti-inflammatory concentration for BSr, providing the most pronounced and balanced suppression of all three inflammatory genes. Of note, the anti-inflammatory performance of BCe at 12.5 and 25 μg/mL was comparable to that of BSi (Figure 4D). When the concentration was increased to 50 μg/mL, its inhibitory effect was markedly enhanced, with *Il6*, *Tnfα* and *Il1β* expression levels reduced by 69.7%, 27.0% and 51.6%, respectively, relative to BSi at the same dose (Figure 4D(a–c)). This potentiated inhibition is consistent with the redox-active properties of CeO_2_ nanocrystals present in BCe.

Based on the assessment of the anti-inflammatory properties of MBGNs doped with four different ions, we concluded that BCa, BSr, and BCe exhibited anti-inflammatory characteristics at appropriate concentrations, surpassing the effects of BSi; conversely, BCo showed a pro-inflammatory effect. When comparing NPs with the same concentration, BSr demonstrated the most anti-inflammatory effect (Supplementary Figure S4A–C).

In order to further investigate the anti-inflammatory mechanism of BCa, BCo, BSr, and BCe, the effect of macrophage phenotype was investigated utilizing flow cytometry (Figure 4E). Compared with the untreated blank control group, the LPS-stimulated group showed a marked increase in CD86 expression (*P* < 0.01), confirming polarization toward the pro-inflammatory M1 phenotype. After treating with BSi, BCa, BCo and BCe, the expressions of CD86 were found to be 56.12%, 57.39%, 57.28% and 60.15%, respectively, which were slightly lower than those of the LPS-induced group, but no difference was observed. The incorporation of Ca, Co and Ce elements did not notably enhance the macrophages’ ability to transition from the M1 to the M2 phenotype. However, it is noteworthy that CD86 expression in the BSr group was markedly reduced compared with the LPS-induced group (Figure 4F). This outcome suggests that BSr has the capacity to effectively regulate the phenotypic transition of macrophages, thereby exerting a degree of inhibition on the inflammatory response.

### Antioxidant properties analysis of MBGNs

**Figure 5:**
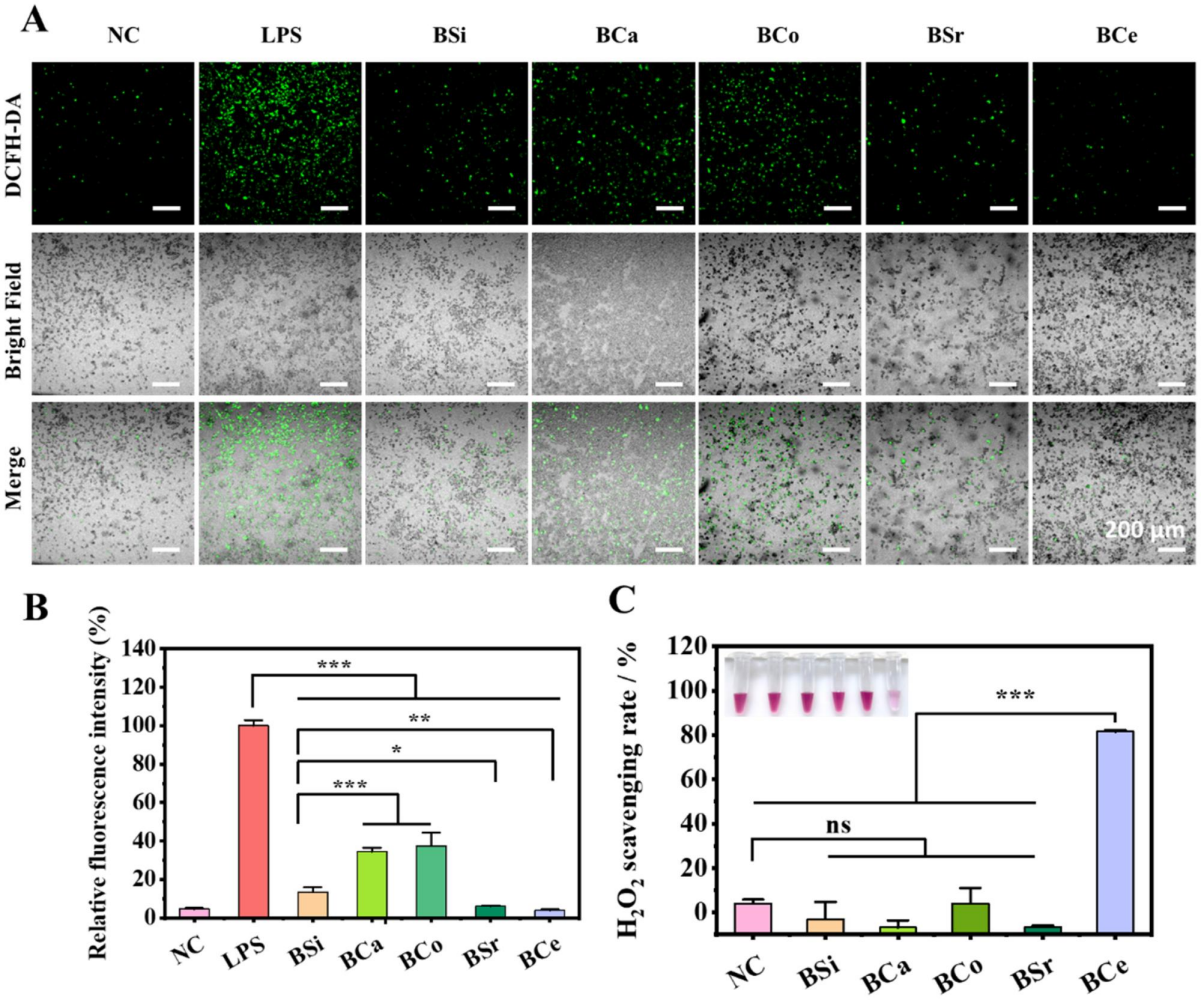
ROS scavenging ability of BSi and MBGNs. (**A**) DCFH-DA fluorescence and bright-field images after RAW264.7 cells are treated with MBGNs. (**B**) Quantitative statistics of fluorescence intensity (*n* = 3, *P < 0.05, **P < 0.01, ***P < 0.001). (**C**) Schematic diagram of antioxidant properties of BCe.

ROS fluorescence imaging (Figure 5A) showed that, compared with the LPS-stimulated control group, all SBGNs markedly reduced intracellular green fluorescence after 24 h of treatment (*P* < 0.001). However, quantitative analysis (Figure 5B) revealed that ROS fluorescence in the BCa and BCo groups was 155.1% and 178% higher than that of BSi, respectively, suggesting that Ca and Co incorporation attenuated the ROS-scavenging capacity of the materials. In contrast, BSr and BCe reduced ROS fluorescence by 54.2% and 71.3%, respectively, relative to BSi, indicating substantially stronger ROS-scavenging activity.

Given the superior antioxidant performance of BCe, we further investigated its redox-regulatory potential. CAT-like assays (Figure 5C) showed that only BCe rapidly catalyzed the decomposition of H_2_O_2_, achieving a removal efficiency of 81.6%, whereas the other samples exhibited negligible activity, thereby confirming that BCe possesses intrinsic CAT-like activity. Overall, incorporation of Ce and Sr into BSi enhanced its antioxidant performance, with BCe in particular exhibiting markedly superior catalytic ROS-scavenging capacity.

### Cell migration and tubular formation induced by MBGNs

**Figure 6:**
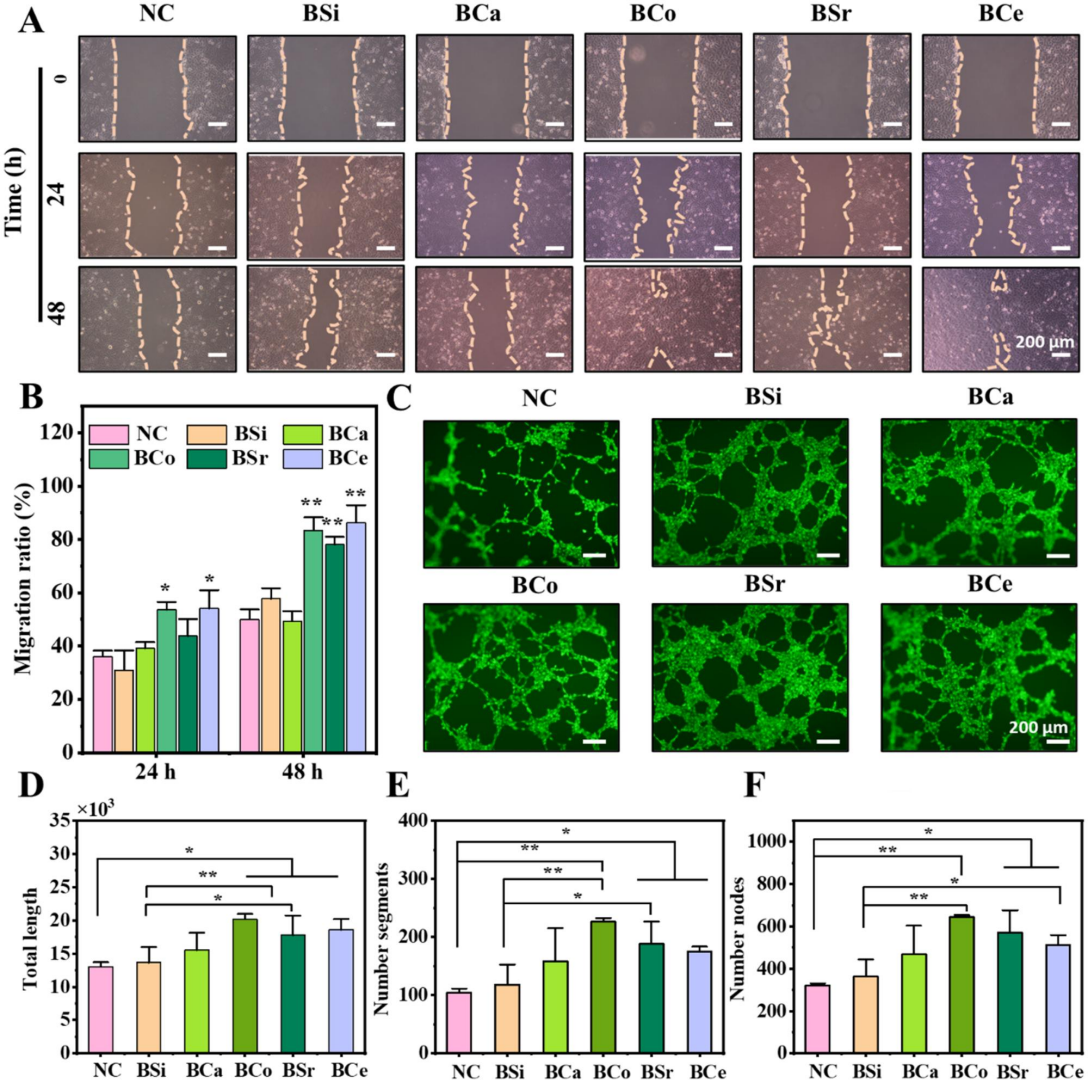
Cell migration and tubulogenesis ability of BSi and MBGNs. (**A**) Migration ability of HUVECs cultured with MBGNs at different time points. (**B**) Quantitative results of migration ability (*n* = 3,*P < 0.05, **P < 0.01). (**C**) Results of MBGNs inducing the formation of vascular networks in HUVECs endothelial cells. Quantitative statistical results of vascular network formation (**D**) Total length, (**E**) Number of segments, and (**F**) Number of meshes (*n* = 3,*P < 0.05, **P < 0.01).

Given the pivotal role of endothelial cell migration in angiogenesis and wound repair, we first used a scratch-wound assay to systematically assess the effects of MBGNs on HUVECs migration. As shown in Figure 6A, after 24 h of treatment, the scratch widths in the BSi and BCa groups were comparable to those in the negative control (NC) group, whereas the gaps in the BCo, BSr and BCe groups were noticeably narrower, indicating a stronger pro-migratory effect. By 48 h, the scratches in the BCo and BCe groups were almost completely closed, demonstrating a pronounced enhancement of cell migration. Quantitative analysis (Figure 6B) revealed that, at 24 h, the migration rates in the NC, BSi, BCa, BCo, BSr and BCe groups were 36.1%, 30.9%, 39.2%, 53.7%, 43.9% and 54.1%, respectively, whereas at 48 h the migration rates in the BSi, BCa, BCo, BSr and BCe groups reached 57.8%, 49.3%, 83.4%, 78.1% and 86.3%, respectively. Notably, at 48 h, the migration rates in the BCo and BCe groups were increased by 33.4% and 36.3%, respectively, relative to the NC group, markedly exceeding those of the other groups and indicating that Co- and Ce-doped SBGNs strongly enhance HUVECs migration.

Building on these findings, we next evaluated the effects of MBGNs on vascular network formation using a Matrigel tubular formation assay (Figure 6C). Compared with the NC group, all SBGNs treated groups promoted the formation of vascular-like networks to varying degrees. Among these, BCo induced the most abundant, well-organized, and structurally complex tubular networks, whereas BCe and BSr also exhibited robust pro-angiogenic activity. By contrast, although BSi and BCa led to modest improvements relative to NC, their overall effects on tube formation remained limited. Quantitative analysis (Figure 6D–F) further revealed that, compared with BSi, BCo produced the greatest enhancement, increasing the number of nodes by 77.3%, the number of segments by 91.8%, and the total tube length by 47.6%. BSr ranked second, with corresponding increases of 57.3%, 59.3% and 29.9%, whereas BCe also yielded substantial improvements of 41.2%, 48.3% and 35.9%, respectively. In contrast, the effects of BCa were similar to those of BSi, with only minor increases across all parameters. These findings suggest that the doping with Co, Ce, Sr and Ca enhances the angiogenic ability of BSi to some extent, and this effect aligns with the trends observed in the migration experiments.

### Angiogenesis capacity evaluation of MBGNs

**Figure 7:**
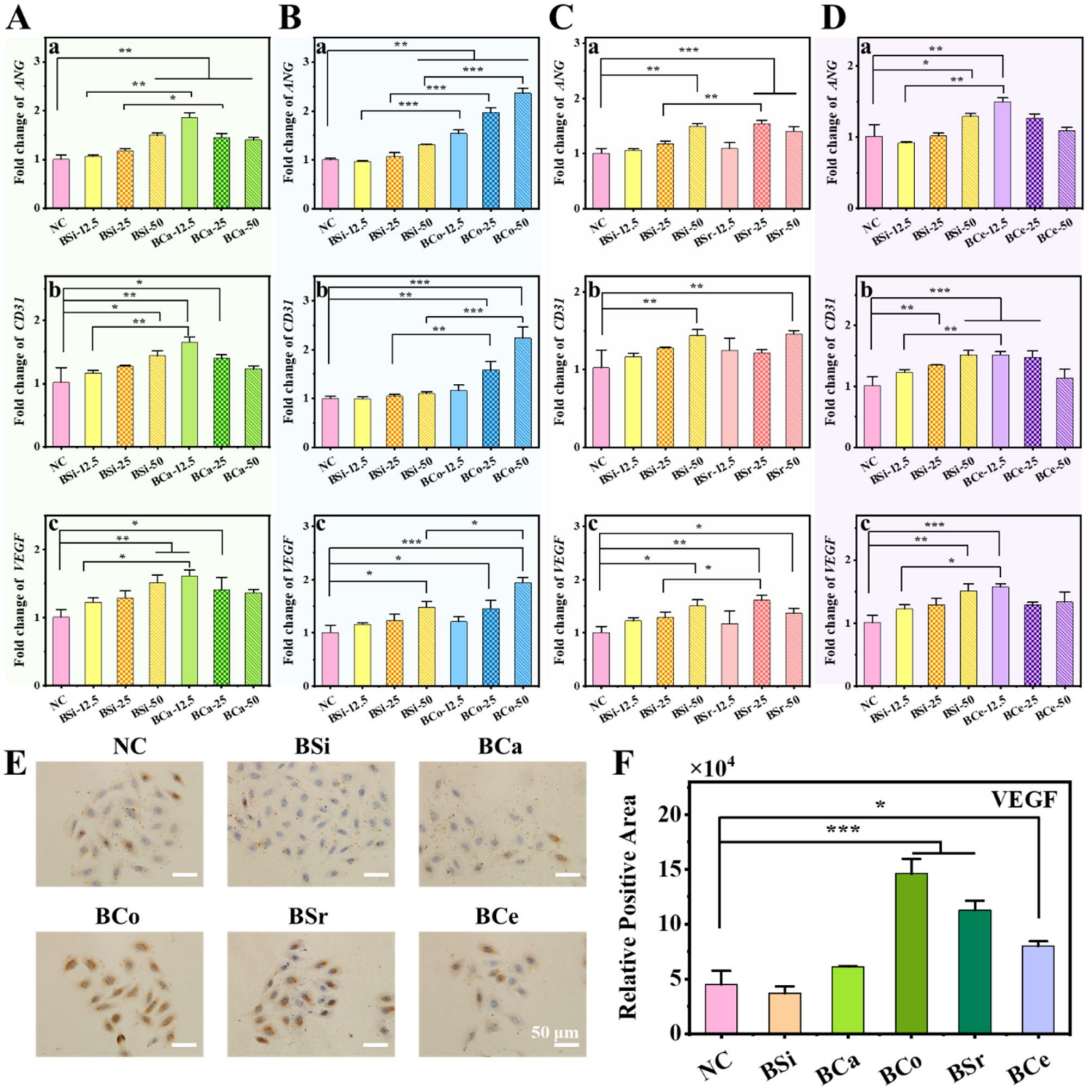
Angiogenesis ability of BSi and MBGNs. (**A–D**) The mRNA expression of (a) *ANG*, (b) *CD31*, and (c) *VEGF* in HUVECs cells with MBGNs: (**A**) BCa, (**B**) BCo, (**C**) BSr, and (**D**) BCe (*n* = 3, *P < 0.05, **P < 0.01). (**E**) Expression of VEGF protein in HUVECs cells after treatment with BSi and MBGNs. (**F**) Statistical diagram of VEGF protein positive signals (*n* = 3, *P < 0.05, ***P < 0.001).

To investigate the pro-angiogenic effects of MBGNs, we examined the mRNA expression levels of *CD31*, *ANG* and *VEGF* in HUVECs after treatment with the different materials (Figure 7A–D). BSi exhibited a clear concentration-dependent effect on the expression of angiogenesis-related genes in HUVECs. At low concentrations, BSi had little effect on the expression of *CD31*, *ANG*, or *VEGF* (Figure 7A(a–c)). When the concentration was increased to 25 μg/mL, *ANG* and *VEGF* were upregulated by 17.4% and 27.9%, respectively, and at 50 μg/mL the expression levels of all 3 genes increased by approximately 40–50%, indicating a marked pro-angiogenic effect. In contrast, BCa enhanced *ANG*, *CD31* and *VEGF* expression already at 12.5 μg/mL, with increases of 68.9%, 38.1% and 60.2%, respectively, all exceeding those of BSi at the same dose (Figure 7A(a–c)). However, no further enhancement was detected at medium or high concentrations, suggesting that the pro-angiogenic effect of BCa is likewise concentration dependent, with a relatively narrow optimal window at low dose.

BCo exhibited pronounced pro-angiogenic activity across all tested concentrations, with a clear dose-dependent enhancement (Figure 7B). At 12.5 μg/mL, the expression of *CD31*, *VEGF* and *ANG* increased by 17.7%, 4.5% and 58.9%, respectively, relative to BSi (Figure 7B(a–c)). At 25 μg/mL, these increments rose to 51.6%, 18.2% and 85.0%. When the concentration reached 50 μg/mL, *CD31*, *VEGF* and *ANG* expression was elevated by approximately 103.2%, 31.4% and 80.8%, respectively, representing the maximal pro-angiogenic response observed for BCo.

The angiogenic effects of BSr were also notable (Figure 7C). At 12.5 μg/mL, BSr exerted only a modest influence on the expression of *ANG*, *CD31* and *VEGF*. Upon increasing the concentration to 25 μg/mL, *ANG* and *VEGF* expression was upregulated by approximately 36.9% and 25.5%, respectively (Figure 7C(a, c)). Further elevation to 50 μg/mL led to higher expression levels of all three genes compared with BSi at the same concentration, indicating that BSr effectively enhances the transcription of angiogenesis-related genes at medium to high doses.

BCe also exhibited distinct pro-angiogenic effects (Figure 7D). At the low concentration of 12.5 μg/mL, BCe increased the transcription of *ANG*, *CD31* and *VEGF* by 62.9%, 23.4% and 28.5%, respectively, compared with BSi at the same dose (*P* < 0.01). At 25 μg/mL, the upregulation of *ANG* (24.3%) and *CD31* (9.9%) persisted but was attenuated, and at higher concentrations the expression of all three genes tended to plateau (Figure 7D(a, b)).

We next evaluated VEGF expression at the protein level by IHC (Figure 7E). Comparative analysis with the blank control group and the BSi group revealed that the BCo and the BSr groups exhibited a higher abundance of brown VEGF-positive signals, indicating a notable angiogenic effect. Conversely, the BCa group and the BCe group displayed relatively fewer positive signals, suggesting a weaker angiogenic effect. Quantitative analysis (Figure 7F) further confirmed that, relative to BSi, the BCa, BCo, BSr and BCe groups increased VEGF protein expression by 65.7%, 294.6%, 204.2% and 116.9%, respectively, in accordance with the gene expression trends observed by RT-qPCR.

### Acute inflammatory wound treatment

**Figure 8:**
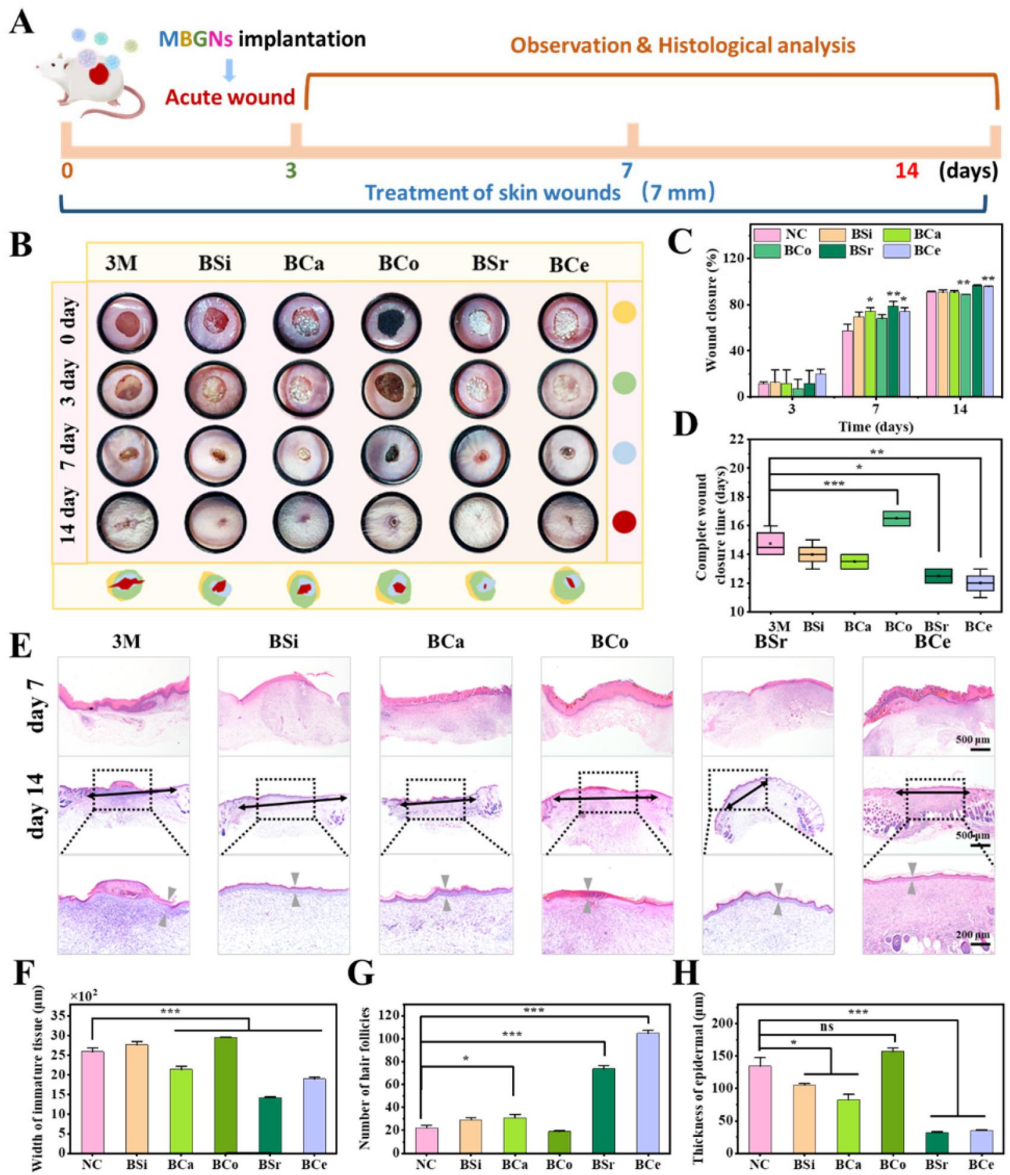
MBGNs promote acute wound healing and regeneration *in vivo*. (**A**) Schematic diagram of the construction and treatment of an acute wound. (**B**) Representative images of the wounds in response to 3M, BSi and MBGNs on days 0, 3, 7, and 14. (**C**) Quantitative analysis of the wound healing rate for each group (*n* = 3, *P < 0.05, **P < 0.01). (**D**) Statistical results of the healing times for each group (*n* = 3, *P < 0.05, **P < 0.01). (**E**) H&E staining of the wound tissue on day 7 and day 14 for each group (the immature tissue and epidermis are marked by black double arrows and gray arrows, respectively). (**F**) Quantitative statistics of the width of immature tissue, (**G**) the number of hair follicles, and (**H**) the thickness of the epidermis (*n* = 3, *P < 0.05, **P < 0.01).

The full-thickness skin defect mouse model was employed to evaluate the wound-healing potential of MBGNs (Figure 8A). On day 3, the BSi-, BCa-, BSr-, and BCe-treated groups all exhibited varying degrees of wound contraction compared with the control group (Figure 8B), with the BCe group in particular showing a more pronounced reduction in wound area (Figure 8C). In contrast, the commercial 3M film group and the BCo group showed minimal macroscopic signs of wound closure. The delayed healing observed in the BCo group may be attributable to the applied powder dose exceeding the biosafety threshold for BCo, thereby inducing cytotoxic effects on wound-resident cells. On day 7, partial closure of the wounds was evident in all experimental groups, with the BSr group demonstrating the most pronounced healing, closely followed by the BCe group. On day 14, wounds in the BSi and BCa groups were essentially closed, although visible scars remained. Notably, wounds in the BSr and BCe groups were completely re-epithelialized and exhibited evident hair regrowth at the original wound sites, indicative of superior wound repair, particularly in the BSr group (Figure 8C). Moreover, we measured the wound-healing times across groups (Figure 8D). The 3M group exhibited a complete healing time of over 15 days, while the BCo group had an extended healing time of 16 days. In contrast, the BSi and BCa groups showed relatively faster healing cycles, with complete wound closure times of 14 and 13 days, respectively, representing a shortened healing cycle compared to the 3M group. Remarkably, the wounds in the BSr group and the BCe group had fully healed by the 12th and 11th days, respectively, which was shorter healing time compared to the other groups. These results clearly demonstrated the substantial acceleration of wound healing by BSr and BCe. Histological analysis (Figure 8E) revealed that during the healing process, the BSr group had formed a more continuous epidermal layer on day 3. In contrast, the re-epithelialization process was essentially completed in the other treatment groups by day 7, accompanied by the formation of neovascularization. By day 14, the wounds in the BSr and BCe groups were completely closed, and the skin appeared intact, with abundant hair follicles, adipocytes, and other appendages. This further substantiates the superior efficacy of BSr and BCe in promoting skin healing and regeneration.

Furthermore, we performed statistical analyses of the width of immature skin tissue, epidermal thickness, and hair follicle counts across the six experimental groups (Figure 8F–H). The results revealed that the BSi-treated group exhibited no statistical difference in immature tissue width compared to the 3M group, measuring 2589 μm and 2767 μm, respectively. However, the wound length in the BCo group was 2958 μm, exceeding that of the 3M group. This outcome may have resulted from the applied BCo concentration exceeding its safety threshold, thereby impairing the healing process. In contrast, the wound lengths in the BCa, BSr and BCe groups measured 2138 μm, 1420 μm, and 1894 μm, respectively. These values indicate that the width of immature tissue was clearly lower than in the 3M and BSi groups. These results suggested that these MBGNs have substantial positive effects on wound healing. Among them, the most pronounced effect was observed in the BSr, followed by the BCe, while the BCa exhibited a relatively weaker effect. Further analysis of epidermal thickness at 14 days revealed that the BCo group and the 3M group had the thickest epidermis, measuring 134 μm and 157 μm, respectively. There was no statistical difference observed. However, the epidermal thickness in the BSi and BCa groups was reduced to 105 μm and 82 μm, respectively, confirming the reparative effect of MBGNs. By contrast, the BSr and BCe groups demonstrated superior efficacy in promoting wound healing, with epidermal thickness measurements of only 32 μm and 34 μm, respectively, approaching the thickness of normal skin tissue. The healing effect of the BSr group was particularly pronounced. Additionally, analysis of hair follicle counts showed that, compared with the 3M blank control group (22 follicles), the BSi, BCa and BCo groups had 29, 31 and 19 follicles, respectively, indicating only minor differences. The BSr and BCe groups showed marked increases, reaching 73 and 104, respectively, with the BCe group displaying the most pronounced effect.

In summary, BSr showed the most effective skin repair outcomes, likely attributable to its strong anti-inflammatory and pro-angiogenic properties. BCe also exhibited notable benefits, primarily through its antioxidant capacity and ability to promote cell migration. In contrast, BCa displayed limited repair potential *in vitro*, possibly because of insufficient ion release. The therapeutic effect of BCo was constrained by potential toxicity at the tested dosage, suggesting the need for further dose optimization and safety evaluation. It should be noted that the acute wound model primarily reflects the baseline repair ability of MBGNs under physiological inflammation, but does not fully replicate the persistent inflammation seen in infected wounds. To further assess their therapeutic potential under pathological conditions and explore the optimized use of BCo, we next established an MRSA-infected wound model for in-depth investigation.

### MRSA-infected inflammatory wound treatment

**Figure 9:**
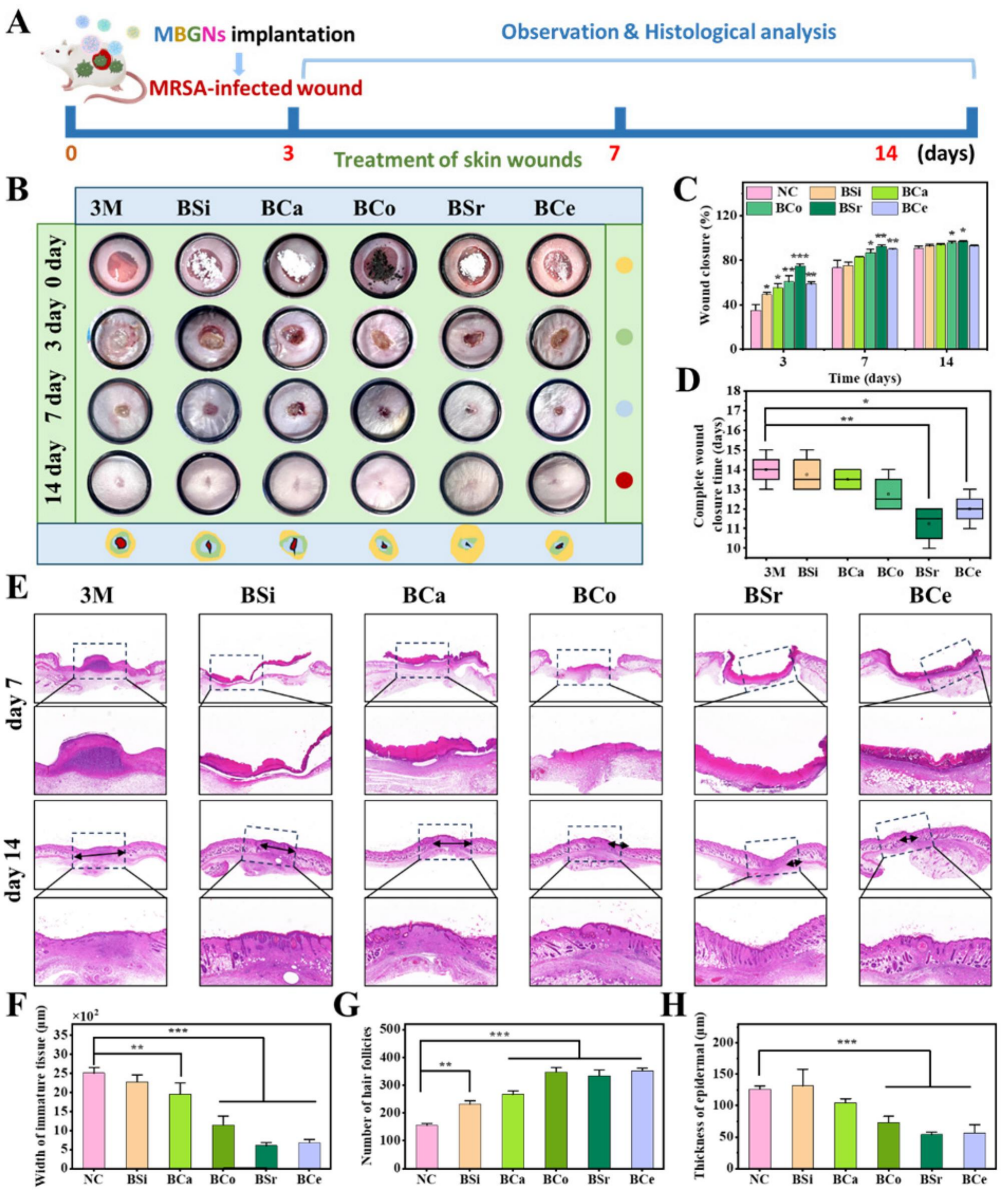
MBGNs promote MRSA-infected inflammatory wound healing and regeneration *in vivo*. (**A**) Schematic diagram of the construction and treatment of an MRSA-infected wound. (**B**) Representative images of the wounds in response to 3M, BSi and MBGNs on days 0, 3, 7, and 14. (**C**) Quantitative analysis of the wound healing rate for each group (*n* = 3, *P < 0.05, **P < 0.01). (**D**) Statistical results of the healing times for each group (*n* = 3, *P < 0.05, **P < 0.01). (**E**) H&E staining of the wound tissue on day 7 and day 14 for each group (the immature tissue is marked by black double arrows). (**F**) Quantitative statistics of the width of immature tissue, (**G**) the number of hair follicles, and (**H**) the thickness of the epidermis (*n* = 3, *P < 0.05, **P < 0.01,***P < 0.001).

To better mimic the pathological features of clinically refractory wounds, we established an MRSA-infected wound model with impaired healing capacity to evaluate the therapeutic effects of different MBGNs (Figure 9A). In this experiment, the BCo dosage was optimized and reduced to minimize adverse effects from excessive ion concentrations and to ensure *in vivo* safety. As shown in Figure 9B and C, the 3M group exhibited slight contraction but severe infection symptoms on day 3, while BSi and MBGN-treated groups accelerated wound closure and reduced inflammation to varying degrees. The BSr group achieved a closure rate of 74%, followed by BCa (55.19%), BCo (60.74%) and BCe (58.53%). On day 7, all treated wounds had further contracted, with BSr showing the greatest reduction and BCe and BCo also displaying clear healing trends. On day 14, BSr and BCo achieved over 95% closure accompanied by hair regrowth, indicating excellent repair. Healing time analysis (Figure 9D) revealed that wounds in the 3M group healed in approximately 14 days, while BSi and BCa shortened this to around 12 days. BCo and BCe completed healing in approximately 12 and 12.5 days, respectively, and BSr achieved the fastest healing at about 11 days. Overall, optimized BCo showed no adverse effects and promoted effective healing, while BSr demonstrated the most potent therapeutic outcome, likely due to its superior anti-inflammatory and angiogenic properties.

Histological analysis (Figure 9E) further revealed the healing process in each group. On day 3, the 3M group had not yet formed a continuous epidermal layer, and numerous of infiltrating inflammatory cells were present in the dermis and wound margins, indicating inadequate infection control. In the BSi and BCa groups, partial re-epithelialization was evident at the wound sites, with reduced inflammation compared to the 3M group, although tissue repair remained incomplete. In the dose-optimized BCo group, no apparent signs of irritation were detected, and its healing-promoting effect was markedly improved compared to the 3M and BSi groups. The BSr and BCe groups exhibited more pronounced re-epithelialization and a marked reduction in inflammatory cells, suggesting that they effectively suppressed inflammation and promoted early tissue repair. By day 14, wounds in the 3M, BSi and BCa groups were largely closed; however, the tissue structure appeared irregular, the epidermis was relatively thick, and newly formed appendages (e.g. hair follicles) were sparse, indicating limited repair quality. In contrast, the BSr, BCo, and BCe groups exhibited a continuous and intact epidermis with thickness close to that of normal skin, accompanied by abundant newly formed hair follicles and appendages in the dermis. Among these, the BSr group demonstrated the best regenerative outcome, followed by BCe and BCo.

To evaluate the quality of tissue repair, we quantitatively analyzed the width of immature skin tissue, epidermal thickness, and hair follicle numbers (Figure 9F–H). The results showed that the immature tissue width in the BSi-treated group was 2281 μm, slightly improved compared with 2507 μm in the 3M group, indicating a modest reparative effect. However, BCa, BCo, BSr and BCe further reduced the wound widths to 1956 μm, 1447 μm, 602 μm and 678 μm, respectively, all of which were lower than those in the 3M and BSi groups. Among them, BSr and BCe exhibited the most pronounced improvement, suggesting accelerated tissue maturation and remodeling, whereas BCo showed a moderate effect, while BCa was relatively weaker. Further analysis of epidermal thickness on day 14 revealed values of 121 μm, 131 μm and 104 μm for the 3M, BSi and BCa groups, respectively, with only limited differences, thereby confirming the baseline reparative effect of MBGNs materials. In contrast, the epidermal thicknesses of the BCo, BSr and BCe groups were 73 μm, 53 μm and 56 μm, respectively, approaching that of normal skin. This demonstrated their advantages in reconstructing epidermal barrier, with BSr showing the most pronounced effect, followed by BCe. Statistical analysis of hair follicle numbers further validated these findings. The control group formed only 155 hair follicles, while the BSi and BCa groups increased to 231 and 266, respectively. Notably, the BCo, BSr and BCe groups showed pronounced increases, reaching 347, 333 and 351 follicles, with BCe displaying the strongest effect.

The results demonstrated that BSr exerted the most pronounced reparative effect in MRSA-infected wounds, whereas the BCe group also exhibited robust pro-healing activity. After dose optimization, BCo treatment effectively shortened the healing time, while BCa showed only modest reparative efficacy. Compared with acute wounds, MBGN treatment in MRSA-infected wounds enhanced wound closure, angiogenesis, and regeneration of skin appendages, thereby improving the overall quality of tissue repair.

## Discussion

In this study, four types of binary silicate-based MBGNs (Bca, Bco, BSr and BCe) were synthesized via a sol-gel method combined with solid-state reaction. All SBGNs exhibited well-defined spherical morphologies and typical mesoporous frameworks, while metal-ion incorporation further modulated the compactness of the mesoporous network and the surface chemical environment. BCa and BSr retained mesoporous core structures similar to BSi, characterized by relatively dense and continuous frameworks. Although Ca^2+^/Sr^2+^ incorporation is theoretically expected to reduce the connectivity of the silicate network, shrinkage during calcination led to overall framework contraction and a slight narrowing of the mesopore channels, reflected by a modest decrease in average pore size [22]. By contrast, BCo and BCe displayed looser mesoporous frameworks and pronounced local structural rearrangements under TEM, indicating stronger perturbation of the silicate network. In particular, branched, dendritic deposits were observed on the surface of BCe particles, which may partially block or fill pore openings and thus further reduce the apparent pore diameter. These structural differences are expected to directly influence cell adhesion, ion exchange, and subsequent cellular responses, and therefore provide a critical structural basis for tissue-repair–related bioactivities [23]. The Ca-, Co- and Sr-doped BGNs remained the amorphous structure, whereas the CeO_2_ nanocrystals present in BCe endowed the material with unique redox properties closely associated with its antioxidant performance [24]. Overall, incorporation of different metal ions modulated the mesoporous framework, local interfacial chemistry, and phase composition of SBGNs, thereby establishing distinct structure–function coupling modes in the context of tissue repair.

For mineralization assays, BCa and BSr showed strong apatite-nucleating ability, suggesting that these dopants decrease the crosslinking of the silicate network, increase the density of surface-active sites, and thereby promote hydroxyapatite nucleation and growth [25, 26]. In contrast, BCo and BCe did not develop pronounced apatite-like phases, indicating relatively lower surface reactivity. Taken together, these mineralization results demonstrate that all SBGNs retain the characteristic interfacial reactivity of MBGNs and provide a fundamental basis for subsequent investigations into their roles in tissue repair.

In addition to the biomineralization activity, BSi, BCa, BSr, and BCe groups exhibit excellent cell compatibility, maintaining high cell viability even at a high concentration of 200 μg/mL. However, BCo exhibited clear cytotoxicity at concentrations exceeding 50 μg/mL, which could be attributed to multiple Co^2+^-induced cytotoxic mechanisms. On one hand, elevated Co^2+^ levels can trigger oxidative stress, leading to mitochondrial dysfunction and cell apoptosis. Additionally, Co^2+^ can catalyze Fenton-like reactions, converting hydrogen peroxide into highly reactive hydroxyl radicals(·OH), thereby exacerbating oxidative damage and ultimately inducing cytotoxicity [27].

Previous studies have indicated that SBGNs can modulate inflammatory responses and regulate macrophage phenotype transformation [1]. In this work, BSr exhibited the most pronounced anti-inflammatory effect: it suppressed the expression of pro-inflammatory genes (*Il6*, *Il1β* and *Tnfα*) and promoted the transition of macrophages from the pro-inflammatory M1 phenotype to the anti-inflammatory M2 phenotype [28, 29], which may be attributed to the ability of Sr^2+^ to suppress NF-κB signaling, thereby reducing the production of pro-inflammatory cytokines [30]. Additionally, Sr^2+^ may regulate calcium-dependent pathways, further facilitating macrophage polarization [28]. In the mouse full-thickness injury model, the strong anti-inflammatory activity of BSr contributed to enhanced tissue repair, consistent with previous research findings. BCa also exhibited moderate anti-inflammatory activity at medium and high concentrations, which may be attributed to Ca^2+^-induced loosening of the silicate network, thereby facilitating the release of metasilicate species with anti-inflammatory effects [31]. In contrast, BCo displayed a pro-inflammatory tendency at higher concentrations, likely due to increased Co^2+^ release and the consequent Fenton-like reactions that drive ROS accumulation [32]. By comparison, BCe primarily modulated inflammation through efficient ROS scavenging mediated by the reversible Ce^3+^/Ce^4+^ redox cycling of CeO_2_ nanocrystals [33].

This study further revealed marked differences in the ROS-scavenging capacities of the various SBGNs, with BCe exhibiting the most pronounced antioxidant behavior. The superior antioxidant performance of BCe is primarily ascribed to the reversible redox activity of CeO_2_ nanocrystals on its surface. The Ce^3+^/Ce^4+^ redox cycle enables continuous capture and conversion of intracellular ROS and catalyzes the decomposition of H_2_O_2_, thereby imparting CAT-like activity [34]. This dynamic redox process helps maintain intracellular redox homeostasis under conditions of elevated oxidative stress and, in parallel, promotes tissue repair by attenuating ROS-mediated inflammatory responses [21]. In contrast, doping with Ca or Co attenuates the ROS-scavenging capacity of the SBGNs: Ca^2+^ may induce intracellular Ca^2+^ overload and disrupt redox balance, whereas Co^2+^ can exacerbate ROS accumulation through Fenton-like reactions, ultimately limiting the antioxidant potential of BCo [35, 36].

Angiogenesis is a critical phase of the wound-healing process. Previous studies have demonstrated that SBGNs can promote angiogenesis by releasing bioactive ions such as silicate (${\text{SiO}}_{3}^{2-}$) upon hydrolysis, thereby modulating key cellular signaling pathways [37]. In this study, BCo exhibited the most pronounced pro-angiogenic effect, which was likely attributable to the ability of Co^2+^ to mimic cellular hypoxia, activate the PI3K/AKT signaling pathway, and regulate the transcriptional activity of hypoxia-inducible factor HIF-1α, thereby promoting neovascularization [38, 39]. In addition, Co^2+^can enhance endothelial cell migration and cytoskeletal remodeling, improve tube-formation efficiency and confer a multi-target, synergistic pro-angiogenic advantage [40]. BSr also displayed strong pro-angiogenic potential at medium and high concentrations, which may be related to Sr^2+^-mediated activation of the Erk1/2 pathway, rendering endothelial cells more responsive to angiogenic cues [41, 42]. By contrast, the pro-angiogenic effect of BCa was relatively moderate and more strongly dose dependent, possibly because Ca^2+^ regulates endothelial cell proliferation and migration via store-operated calcium entry (SOCE) channels, thereby modulating the expression of angiogenesis-related genes [43]. Moreover, the CeO_2_ nanocrystals in BCe may further promote angiogenesis by enhancing p38 MAPK activation [44].

In the acute wound model, BSr exert anti-inflammatory, pro-angiogenic, and macrophage-modulating effects that provide strong biological support for tissue regeneration. These regulatory actions alleviate inflammation-induced suppression of hair follicle stem cell activity and improve the perivascular blood supply around hair follicles, thereby increasing hair follicle density [45, 46]. BCe NPs, with their unique antioxidant properties and enhanced cell migration capacity, protect hair follicle stem cells from oxidative damage and facilitate the aggregation and differentiation of hair follicle-related cells, thus promoting wound repair [47]. By contrast, effective ion release from BCa is relatively low, which limits its capacity to modulate inflammation and cellular activity, whereas the biological behavior of BCo is highly dose dependent; once its biosafety threshold is exceeded, Co-containing particles may impose an additional burden on the surrounding tissues.

Compared with acute wounds, MRSA-infected wounds are characterized by a higher inflammatory burden and sustained bacterial stimulation, which amplifies material-dependent differences in tissue repair. In this complex setting, the inflammation-regulatory and pro-angiogenic pathways elicited by BSr are more readily engaged, allowing more effective modulation of the local wound microenvironment. BCe, through its enzyme-mimetic catalytic activity, efficiently scavenges ROS, attenuates cellular damage, and promotes the regeneration of skin appendages. When appropriately dose-optimized, BCo avoids toxic accumulation and yields more stable reparative outcomes than in the acute wound model. By contrast, the regulatory capacity of BCa remains limited under infectious conditions, as its ion release does not reach levels sufficient to adequately modulate inflammation and oxidative stress.

Although our study successfully developed binary BG nanosystems doped with Ca, Co, Sr, and Ce, and investigated their biological properties, such as cytotoxicity, anti-inflammatory, antioxidant, and angiogenesis capabilities, there are certain limitations to our study. For instance, while BCe have been reported to possess antibacterial properties, we did not confirm this in our study [48, 49]. This discrepancy may be attributed to the doping ratio of metal ions and the synthesis method employed, underscoring the need for further exploration and refinement. Furthermore, although BCo exhibited pronounced pro-angiogenic and cell migration capabilities *in vitro*, its efficacy in acute wound repair was limited by the safe dosage range, failing to sufficiently promote tissue remodeling and even causing delayed healing at high doses. Notably, in the MRSA infection model, dosage optimization avoided these adverse effects, and BCo showed some potential in promoting wound closure, although its overall efficacy remained inferior to that of BSr and BCe. Therefore, comprehensive research focusing on the biosafety and modification of BCo is urgently required. Lastly, although the BSr group exhibited promising results in reducing the expression of inflammatory factors and regulating macrophage phenotype transformation, the role of other immune cells, such as lymphocytes, dendritic cells, and neutrophils, in the wound healing process remains unclear. These aspects necessitate further investigation in future research endeavors. Although we just chose the four metal-based BGN for comparison in different biological properties, they should represent a class of metal ions related to tissue repair. Sr, Co, Ce should have similar properties with alkaline earth metals (like Mg) [50, 51], transition metals (like Mn, Fe, Cu, and Zn) and rear earth elements (like La and Eu) [52–54].

## Conclusion

In this study, the binary silicate-based MBGNs (BCa, BCo, BSr and BCe) could be synthesized via a sol–gel/solid-state strategy for systematically evaluating their bioactivities in wound repair. BCa demonstrated moderate bioactivity, BSr showed potent anti-inflammatory and immunomodulatory effects, BCe exhibited robust antioxidant capacity with catalase-mimicking activity, and BCo enhanced angiogenesis in a dose-dependent fashion. In acute wounds, BSr and BCe accelerated closure and remodeling, whereas BCa exerted weaker effects and BCo delayed healing at higher doses. In MRSA-infected wounds, the multifunctional advantages of BSr and BCe such as immune regulation, oxidative stress alleviation and angiogenesis were further amplified, leading to rapid and high-quality regeneration. Notably, optimized BCo achieved moderate efficacy, whereas BCa remained limited. Collectively, these findings demonstrate that simplified binary systems enable single-ion doping to directionally regulate bioactivity with distinct functional outcomes, providing a rational design strategy for functional SBGNs and underscoring the translational promise of MBGNs for both acute and infected wound repair.

## Supplementary Material

rbag004_Supplementary_Data
