# Supplementary material for: Calcium, cobalt, strontium and cerium-based binary silicate bioactive nanoglasses: rational comparison in bioactive properties for optimizing tissue repair applications
Source: Regen Biomater. 2026 Mar 1;13:rbag004. doi: 10.1093/rb/rbag004 (PMC13176458; doi:10.1093/rb/rbag004)
Supplement: rbag004_Supplementary_Data [file rbag004_supplementary_data.docx]

**Supporting information**

**Calcium, Cobalt, Strontium, and Cerium-Based Binary Bioactive Glass Nanoparticles: Rational Comparison in Bioactive Properties for Optimizing Tissue Repair Applications**

Yanzi Zhao ^a #^, Jing Tian ^a #^, Liuyang Zhang ^a^, Long Zhang ^b^, Qian Huang ^a*^, Xiaoyan Qu ^a^, Bo Lei ^a, b, c^*

*^a^ Frontier Institute of Science and Technology, Key Laboratory of Shaanxi Province for Craniofacial Precision Medicine Research, College of Stomatology, Xi’an Jiaotong University, Xi’an, 710054, China*

*^b^ Department of Respiratory and Critical Care Medicine, The Second Affiliated Hospital of Xi’an Jiaotong*

*University, Xi’an, 710004, China*

*^c^ State Key Laboratory for Mechanical Behavior of Materials, Xi’an Jiaotong University, Xi’an, 710054,*

*China*

^*^*Corresponding author*

*E-mail: rayboo@xjtu.edu.cn*

*^#^* *These authors contributed equally to this work.*

**Synthesis Methods**

**1. Materials**

The following chemicals and reagents were procured from specific sources: cetyltrimethylammonium bromide (CTAB), triethanolamine (TEA), tetraethyl orthosilicate (TEOS), calcium nitrate tetrahydrate, strontium nitrate, cerium nitrate hexahydrate, cobalt nitrate anhydrous, and 2'-7'-dichlorodihydrofluorescein diacetate (DCFH-DA) were obtained from Sigma-Aldrich (Darmstadt, Germany). Cyclohexane and ethanol were sourced from Guanghua, China. Phosphate buffer solution (PBS), Dulbecco's Modified Eagle Medium (DMEM), and the Alamar Blue kit were acquired from Gibco Invitrogen, France. Fetal bovine serum (FBS) was purchased from Biological Industries (BI). The cell lines employed in this study were obtained from the cell bank of the Chinese Academy of Sciences.

**2. Preparation of BCa Nanoparticles**

BCa nanoparticles were prepared using BSi as a template via a solid-state reaction method. The process involved dissolving 0.4858 g of Ca(NO_3_)_2_ in 20 mL of ethanol and then adding 1 g of BSi (100% SiO_2_) and stirring for 6 h. The suspension was continuously stirred at room temperature until the ethanol was completely evaporated, resulting in the precursor (BSi loaded with Ca(NO_3_)_2_·4H_2_O). The collected precursor powder was calcined at 600°C for 5 h to obtain BCa nanoparticles.

**3. Preparation of BSr Nanoparticles**

Due to the property of Sr(NO_3_)_2_ being soluble in water and slightly soluble in ethanol, 0.4353 g of Sr(NO_3_)_2_ was dissolved in a mixed solution of 20 mL of water and ethanol (in a 1:1 ratio), followed by the addition of 1 g of BSi powder. The mixture was stirred at room temperature for 9 h until the solution was completely evaporated, yielding the precursor powder (BSi loaded with Sr(NO_3_)_2_). The collected precursor powder was calcined at 600°C for 5 h to obtain BSr nanoparticles.

**4. Preparation of BCe Nanoparticles**

The preparation of BCe nanoparticles was carried out using the same method as described above. Initially, 0.8933 g of Ce(NO_3_)_3_·6H_2_O was completely dissolved in a 20 mL ethanol solution, followed by the addition of 1 g of BSi nanoparticle powder and stirring at room temperature for 6 h until the ethanol was completely evaporated. This resulted in the precursor loaded with BSi and Ce(NO_3_)_3_·6H_2_O, which was then calcined at 600°C for 5 h to yield BCe nanoparticles.

**5. Characterization**

The morphological characteristics of the five nanoparticles were examined using a transmission electron microscope (TEM, HT-7700). The distribution of each metal element and the extent of effective doping in the nanoparticles were observed and semi-quantified using a scanning electron microscope (SEM, QUANTA FEG250) in combination with an energy dispersive spectrometer (EDS, Oxford X-MaxN). Nitrogen adsorption–desorption isotherms were measured using a fully automatic surface area and porosity analyzer (MicrotracBEL, BELSORP-Max II) to characterize the mesoporous structural features of BSi and MBGNs. The crystallographic and chemical structures of the nanoparticle samples were analyzed using an X-ray diffractometer (XRD, d8advance) and a total reflection Fourier infrared spectrometer (FTIR, Nicolet 6700), respectively. Furthermore, X-ray photoelectron spectroscopy (XPS, ESCALAB-Xi+) measurements were conducted using an X-ray spectrometer to determine the elemental compositions and valence states present on the surfaces of the five different nanoparticle samples. Additionally, the nanoparticles were introduced into a pH 7 buffer, and the zeta potential of the nanoparticles was measured.

**6. In vitro biological activity assay**

30 mg of BSi or each MBGNs sample was dispersed in 20 mL of SBF to obtain a suspension with a mass concentration of 1.5 mg/mL, and incubated on a shaker at 37°C and 100 rpm for 7 days. Throughout the immersion process, the suspensions were kept in sealed centrifuge tubes without SBF replacement in order to mimic a relatively closed environment for prolonged ion exchange and mineralization. After incubation, the samples were collected by high-speed centrifugation, treated with acetone to fix the surface structure, rinsed with deionized water to terminate any ongoing reactions, and then dried at room temperature. Finally, XRD was performed on the treated samples to assess the formation of hydroxyapatite (HA) mineral layers on their surfaces.

**7. Cytocompatibility analysis of MBGNs**

To assess the *in vitro* cytocompatibility of the five nanoparticle samples, macrophages (RAW264.7), human umbilical vein endothelial cells (HUVECs), and fibroblasts (L929) were chosen as model cell lines. The cell lines were cultured in DMEM medium (Invitrogen) supplemented with 10% fetal bovine serum (FBS, Invitrogen). RAW264.7, HUVECs, and L929 cells were seeded in 96-well plates at initial densities of 5000, 3000, and 2000 cells/well, respectively. After 24 h of incubation, the cells were co-cultured with BGNs at concentrations of 50, 100, 150, and 200 μg/mL in DMEM at 37°C for 72 h. Cells treated with PBS served as the control, and five replicate treatments were performed for each condition. The viability of the cells was assessed at 24 and 72 h using the AlamarBlue kit. Additionally, the cytocompatibility of the five SBGNs was evaluated using RAW264.7 and HUVECs. To prepare the extracts, SBGNs were immersed in DMEM at a stock concentration of 5 mg/mL and incubated at 37°C for 24 h. The collected supernatants were filtered and diluted with DMEM by factors of 100, 50, 33.3, 25, and 2.5 to achieve final working concentrations of 50, 100, 150, 200 μg/mL, and 2 mg/mL, respectively. Subsequently, cells were treated with these extracts for 24 h, and cell viability was assessed.

**8. Antioxidant properties of MBGNs**

The ability of MBGNs to clear reactive oxygen species (ROS) was assessed using RAW264.7 cells. The cells were seeded on slides in a 24-well plate at a density of 5×10^4^ cells per well and allowed to adhere. Subsequently, the cells were treated with 400 ng/mL LPS and incubates at 37 °C, 5% CO_2_ incubator for 24 h to induce ROS production. After stimulation, a control group (treated with PBS) and experimental groups (treated with 50 μg/mL BSi or MBGNs) were established and incubated for another 24  h. Cells were then stained with a reactive oxygen species detection kit known as DCFH-DA (2 μM). Confocal laser microscopy (FV1200, Olympus) was used to capture images of the stained cells.

Table S1. Chemical Composition and Synthesis Parameters of BSi and MBGNs

| Sample | SiO_2_ (mol%) | Metal oxide | Doping ratio (mol%) | Precursor compound | Appearance/color |
| --- | --- | --- | --- | --- | --- |
| BSi | 100 | - | - | TEOS | White powder |
| BCa | 70 | CaO | 30 | Ca(NO_3_)_2_·4H_2_O | Off-white powder |
| BCo | 70 | CoO | 30 | Sr(NO_3_)_2_ | Off-white powder |
| BSr | 70 | SrO | 30 | Co(NO_3_)_2_·6H_2_O | Dark purple powder |
| BCe | 70 | CeO_2_/Ce_2_O_3_ | 30 | Ce(NO_3_)_3_·6H_2_O | Light-yellow powder |

Table S2. qPCR primer sequence of inflammation- and angiogenesis-related genes

| Genes | species | Forward Primer | Reverse Primer |
| --- | --- | --- | --- |
| *Gapdh* | Mouse | AGCTTAGGTTCATCAGGTAAACTCA | CTGGAACATGTAGACCATGTAGTTG |
| *Tnfa* | Mouse | GATTTGCTATCTCATACCAGGAGAA | ACAGAGCAATGACTCCAAAGTAGAC |
| *Il1β* | Mouse | AAACGGTTTGTCTTCAACAAGATAG | ATTCCATGGTGAAGTCAATTATGTC |
| *Il6* | Mouse | TCTGTAGCTCATTCTGCTCTGG | CCAAGAAGGCAACTGGATGG |
| *GAPDH* | Human | TAGAAAAACCTGCCAAATATGATGA | ATACCAGGAAATGAGCTTGACAAAG |
| *CD31* | Human | AAGAAAAGCAACACAGTCCAGATAG | CAGGATCATTTGAGTTCTTGGTACT |
| *VEGF* | Human | CTGATGAGATCGAGTACATCTTCAA | CTTGTCTTGCTCTATCTTTCTTTGG |
| *ANG* | Human | GATAACTCCAGGTACACACACTTCC | CTTAGGTTTTCTCTGTGAGGGTTTC |


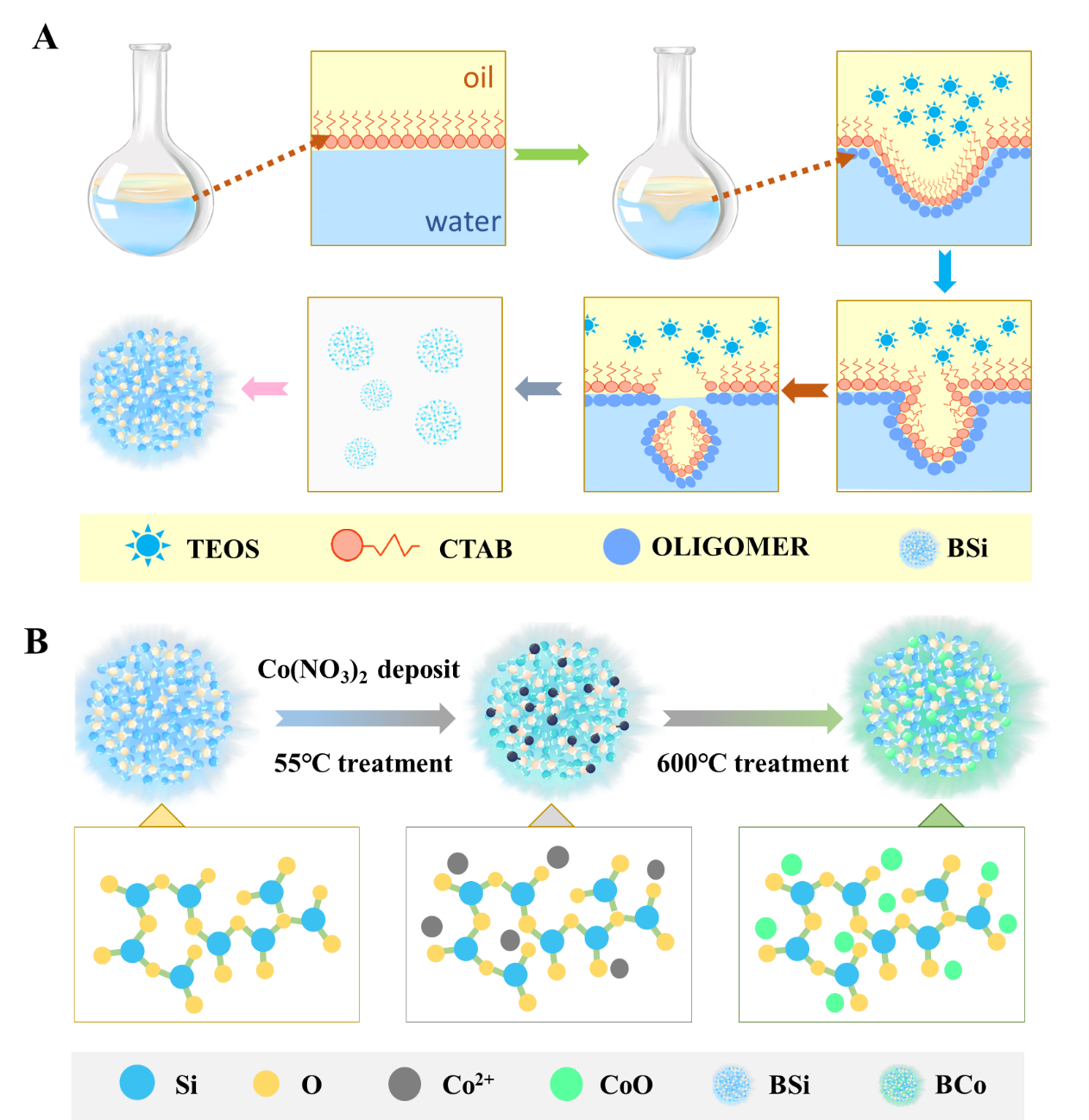


**Figure S1. Schematic diagrams of the formation mechanisms of BSi and MBGNs.** (A) Schematic diagram of the formation mechanism of BSi. (B) Schematic diagram of the formation of BCo.


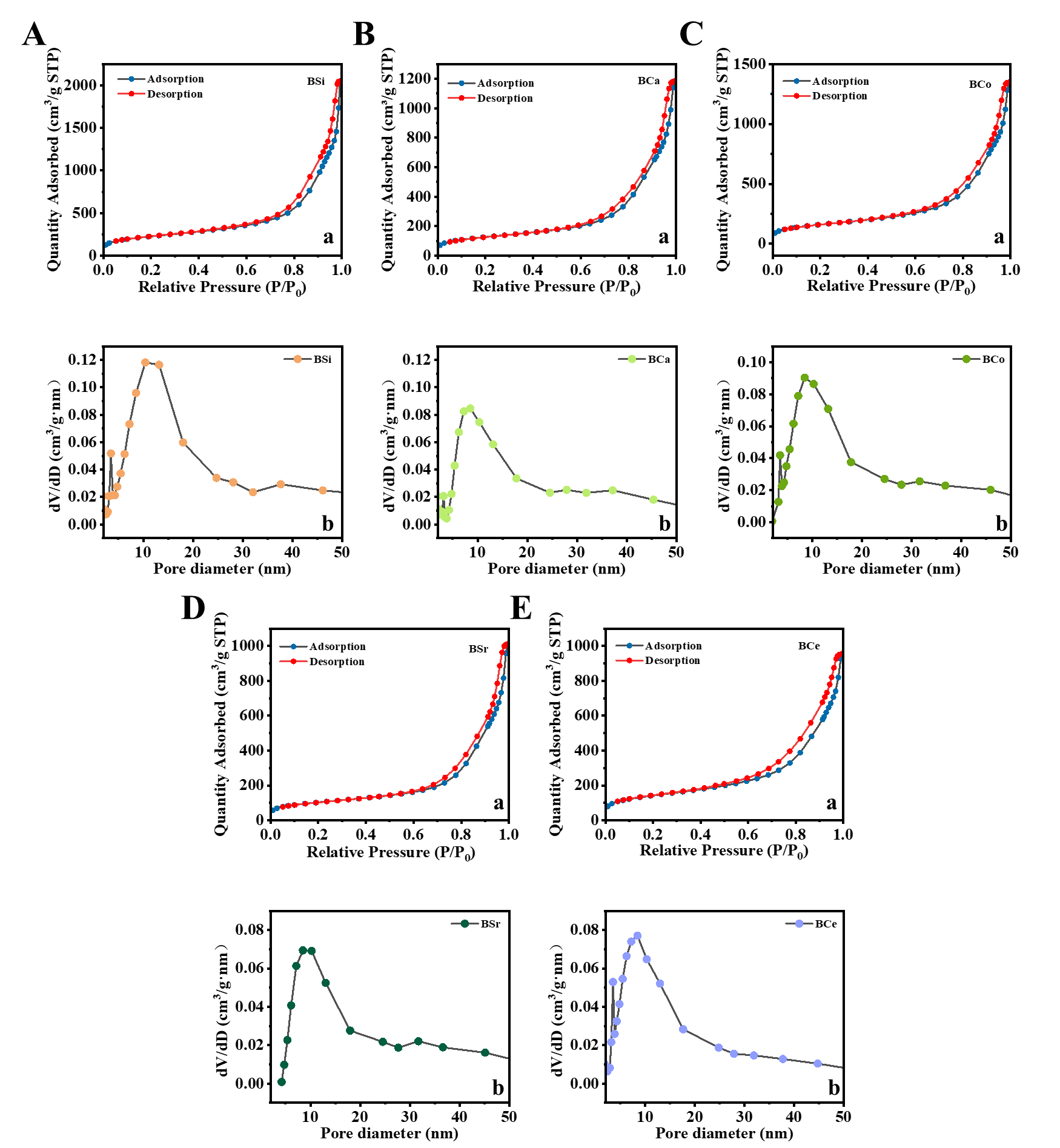


**Figure S2.** Nitrogen adsorption‒desorption isotherms (a) and the corresponding pore size distribution profiles (b) of BSi(A), BCa(B), BCo(C) , BSr(D) and BCe(E) .

**
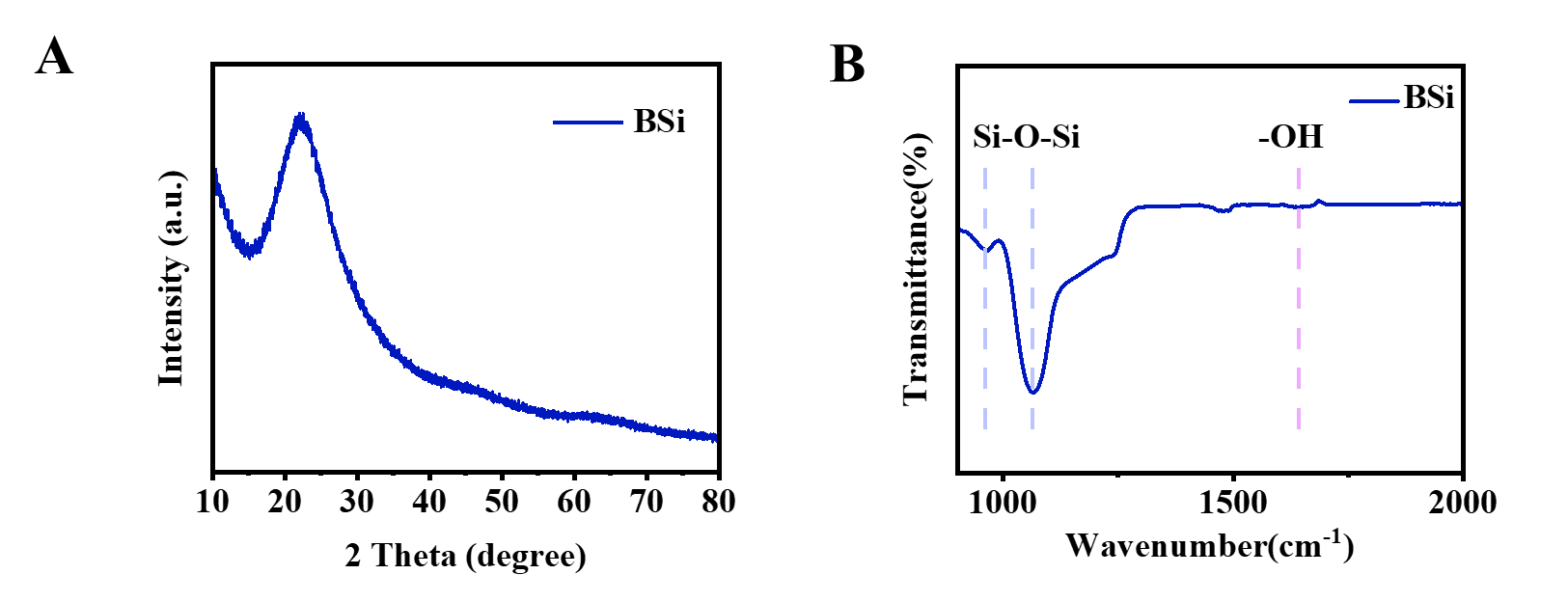
**

**Figure S3.** (A) XRD spectrum analysis of BSi. (B) FTIR pectrum analysis of BSi


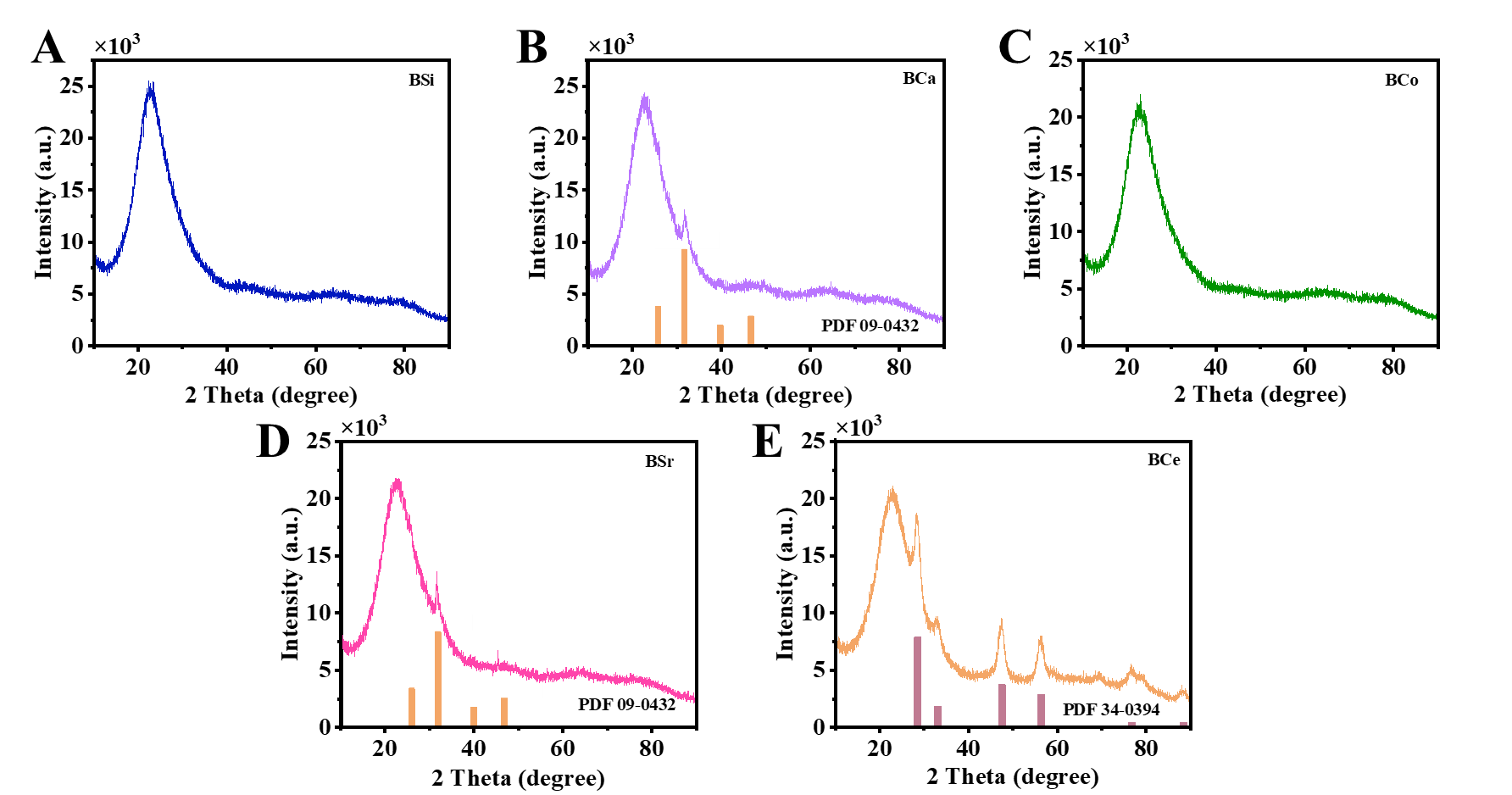


**Figure S4**. XRD patterns illustrating the mineralization behavior of (A) BSi, (B) BCa, (C) BCo, (D) BSr, and (E) BCe after SBF immersion.

**
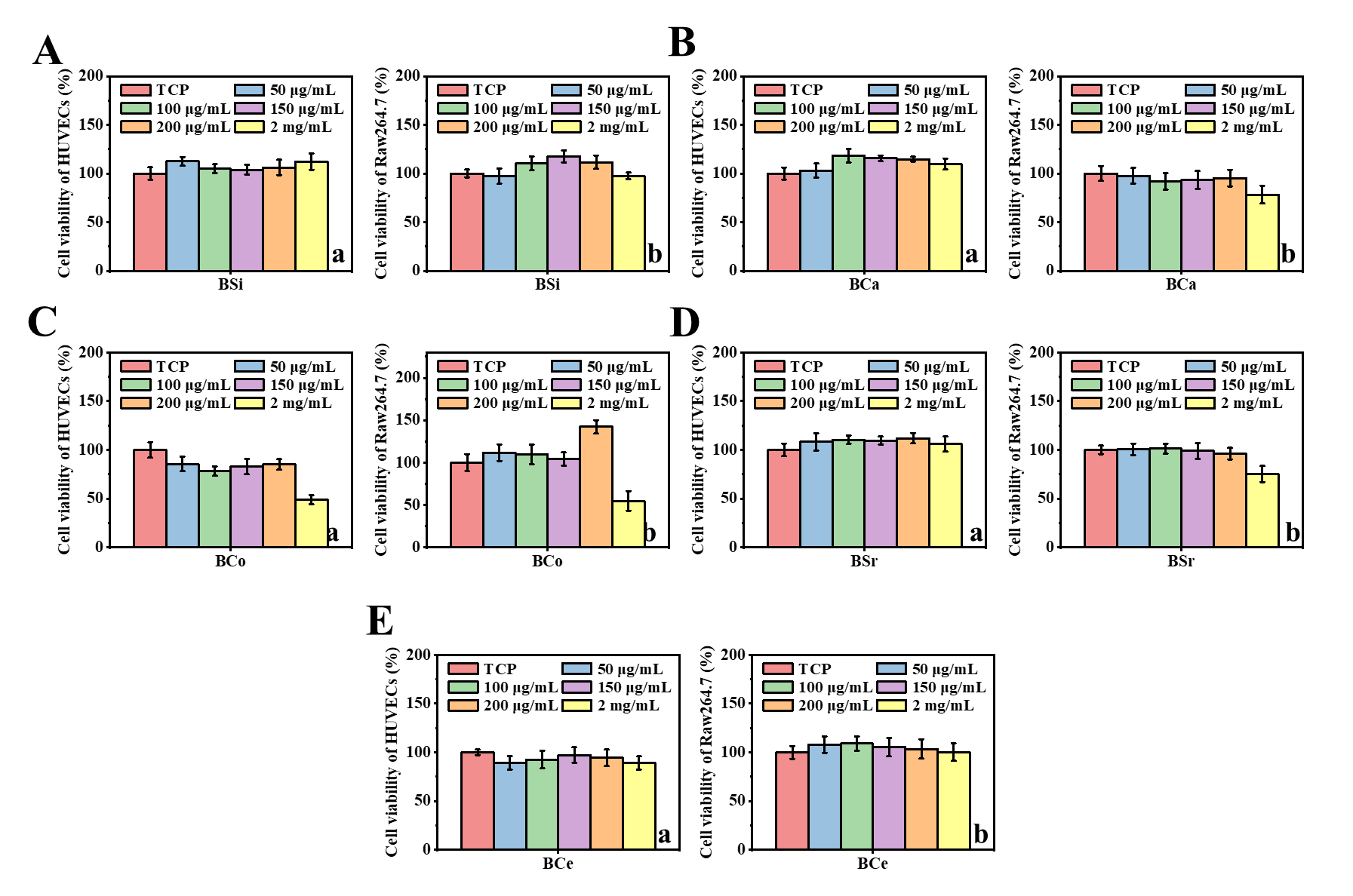
**

**Figure S5. Cytocompatibility of the leach solutions of MBGNs.** (A–E) Cell viability of HUVECs (a) and RAW264.7 (b) cells cultured with leach solutions of BSi (A), BCa (B), BCo (C), BSr (D), and BCe (E).


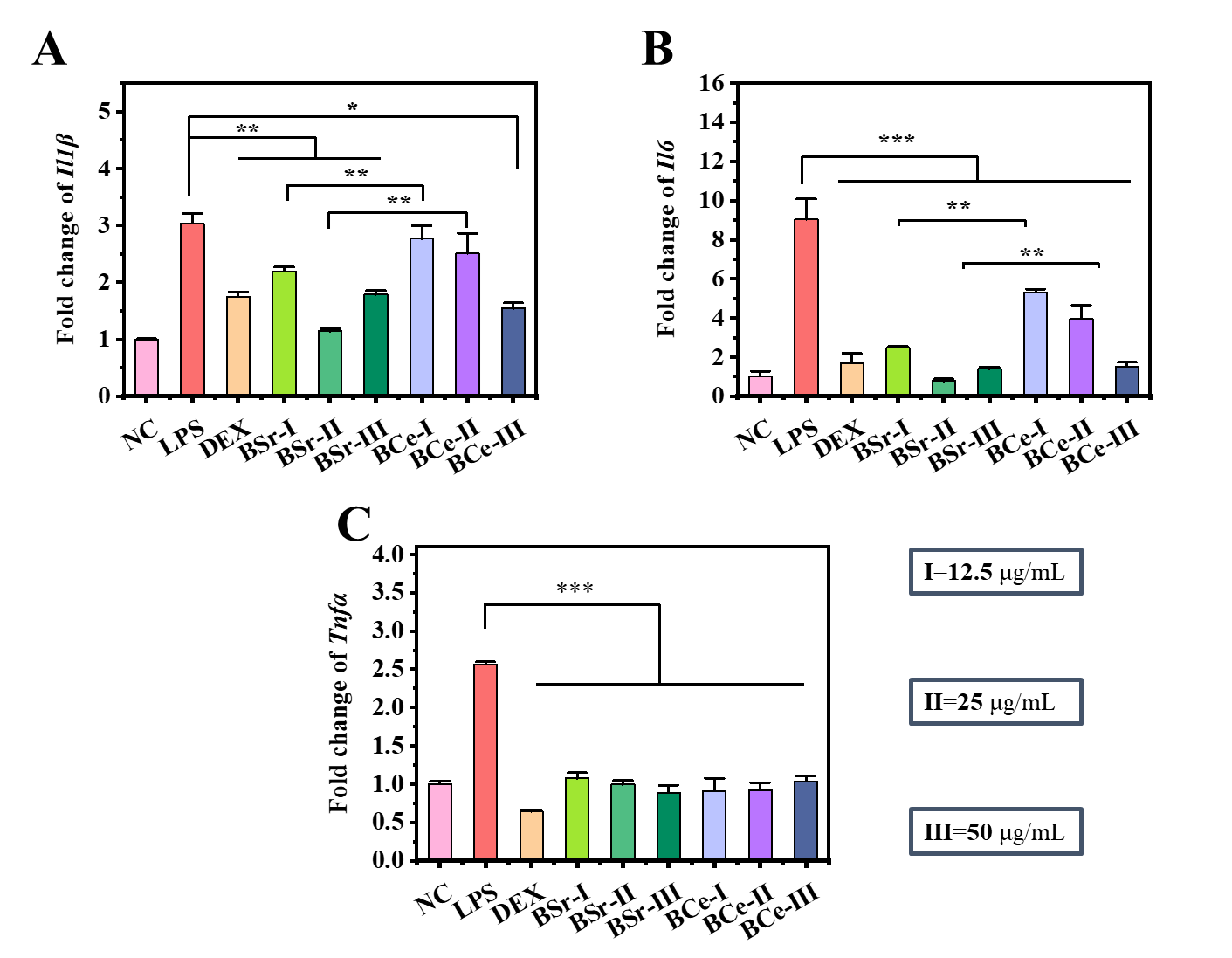


**Figure S6.** Expression of *Il1β* (A), *Il6* (B) and *Tnfa* (C) genes in RAW264.7 cells treated with BSr and BCe.
